# New Phragmalin-Type Limonoids from *Chukrasia tabularis* and Their α-Glucosidase Inhibitory Activity

**DOI:** 10.3390/molecules21010058

**Published:** 2016-01-05

**Authors:** Jun-Lin Peng, Jun Wang, Fan-Dong Kong, Zi-Qi Liu, Pei Wang, Cui-Juan Gai, Bei Jiang, Wen-Li Mei, Hao-Fu Dai

**Affiliations:** 1 Key Laboratory of Biology and Genetic Resources of Tropical Crops, Ministry of Agriculture, Institute of Tropical Bioscience and Biotechnology, Chinese Academy of Tropical Agricultural Sciences, Haikou 571101, China; peng900923@163.com (J.-L.P.); wanghuanlong@163.com (J.W.); kongfandong@itbb.org.cn (F.-D.K.); wangpei@itbb.org.cn (P.W.); narutocn118@163.com (C.-J.G.); 2 College of Pharmacy and Chemistry, Dali University, Dali 671000, China; xiguaxue@163.com (Z.-Q.L.); dalinorthjiang@163.com (B.J.)

**Keywords:** *Chukrasia tabularis*, Meliaceae, limonoid, α-Glucosidase inhibition activity

## Abstract

Phytochemical investigation on the stems of *C. tabularis* led to the isolation of five new phragmalin-type limonoids and six known ones. The structures of the new compounds **1**–**5**, named chukbularisins A–E, were elucidated by spectroscopic techniques (IR, HRESIMS, 1D and 2D NMR) and comparisons with published data. All the compounds were evaluated for *in vitro* α-glucosidase inhibitory activity. Compounds **2**, **3**, **4**, **5**, and **8** exhibited inhibitory activity against α-glucosidase with IC_50_ values of 0.06 ± 0.008, 0.04 ± 0.002, 0.52 ± 0.039, 1.09 ± 0.040, and 0.20 ± 0.057 mM, respectively (using acarbose as positive control, IC_50_ 0.95 ± 0.092 mM).

## 1. Introduction

The genus *Chukrasia* (Meliaceae) comprising only *Chukrasia tabularis* A. Juss and *Chukrasia tabularis* var. *velutina*, which are mainly distributed in the tropical areas of Asia, such as India, Malaysia, and southern China [1]. *C. tabularis* is a timber tree, which is widely cultivated in southern China for the use of urban afforestation and pot culture because it is an evergreen tree. Additionally, its root bark has been used for a long time as a traditional medicine for dispelling wind and heat from the body by the peoples in the tropical areas of Asia [2]. Previous phytochemical studies have reported a number of phragmalin-type limonoids from this plant [3], such as normal phragmalins and their orthoesters, 13/14/18-cyclopropanyl phragmalin-type orthoesters, C(15)-acyl phragmalins, 16-dinorphragmalins, C(15)-acyl 16-dinorphragmalins, 19-dinorphragmalins, and 16,19-dinorphragmalins [4,5,6,7,8,9,10,11,12,13,14,15], and their interesting biological properties including insecticidal, cytotoxic, anti-inflammatory, and delaying of rectifier (*I*_k_) k^+^ current [16,17,18,19,20,21].

**Figure 1:**
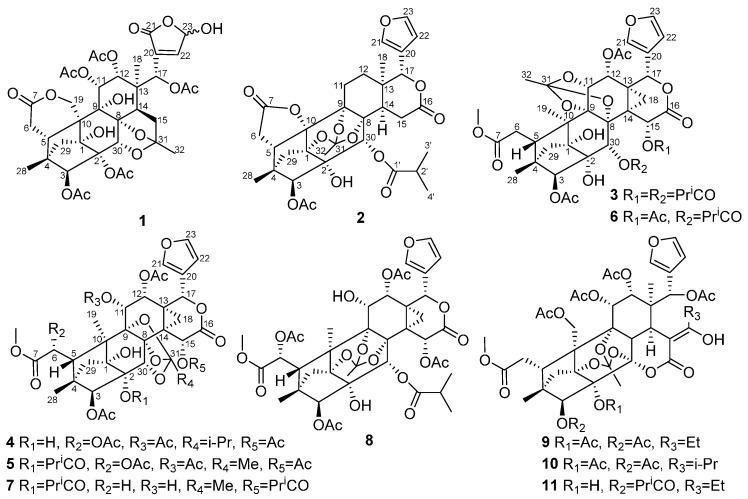
Structures of compounds **1**–**11**.

This study was focused on the isolation and identification of new bioactive limonoids from *Chukrasia tabularis* A. Juss. Bioactivity screening indicated that the EtOAc-soluble extract of the stems of *C. tabularis* showed significant α-glucosidase inhibitory activity. Subsequent chemical investigation led to the identification of five new phragmalin-type limonoids **1**–**5** that we have named chukbularisins A–E, along with six known analogues **6**–**11** (Figure 1). Compounds **2**, **3**, **4**, **5**, and **8** showed inhibitory activities against α-glucosidase. To the best of our knowledge, the α-glucosidase inhibitory activity *in vitro* of limonoids has not yet been reported before. We report herein the isolation, structural elucidation as well as the α-glucosidase inhibitory activity evaluation of eleven limonoids from *C. tabularis*.

## 2. Results and Discussion

**Figure 2:**
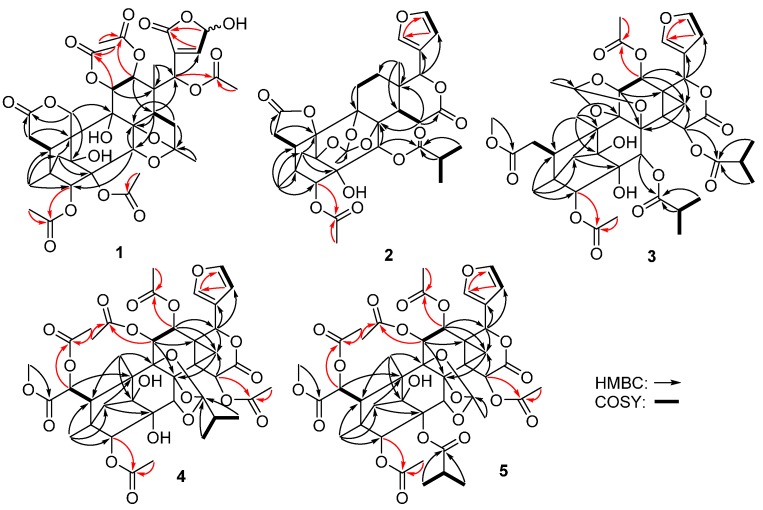
Key HMBC and ^1^H-^1^H COSY correlations for compounds **1**–**5**.

**Figure 3:**
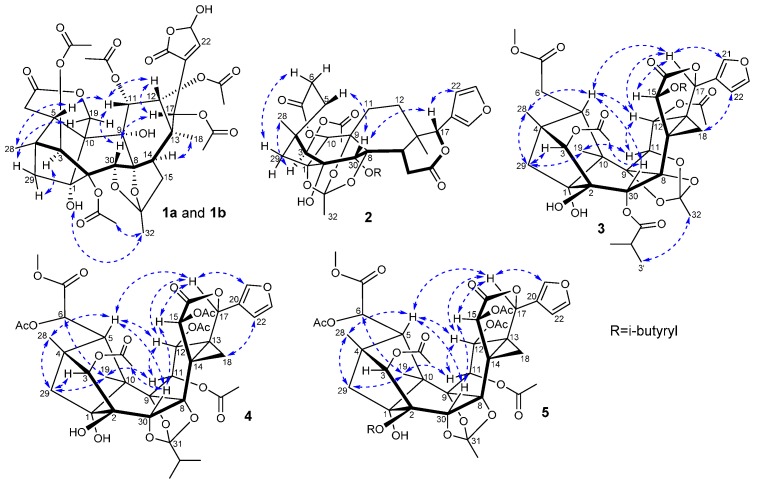
Key ROESY correlations for compounds **1**–**5**.

The EtOAc-soluble extract of the stems of *C. tabularis* was subjected to repeated column chromatography to afford five new phragmalin-type limonoids **1**–**5**, and six known analogues **6**–**11** (Figure 1). Compound **1** was obtained as a white amorphous powder. Its molecular formula was established as C_37_H_44_O_19_ from a pseudomolecular ion peak at *m/z* 810.2817 ([M + NH_4_]^+^ calcd. 810.2815) in the HRESIMS, indicating 16 degrees of unsaturation. The IR spectrum showed hydroxyl group (3443 cm^−1^), carbonyl group (1746 cm^−1^), and olefinic bond (1636 cm^−1^) absorption bands. The ^1^H- and ^13^C-NMR spectra of **1** showed two sets of resonances with a ratio of 3:2 for isomers **1a** and **1b**. The ^1^H-NMR, ^13^C-NMR along with the HSQC data of the major isomer **1a** revealed the presence of two angular methyls (δ_H_ 1.02, 0.90; δ_C_ 18.8, 15.2), five acetoxyls, and typical CH_2_-29 signals of a 4,29,1-ring-bridge [δ_H_ 2.09 and 2.02; δ_C_ 38.9]. Furthermore, the acetoxyls at C-3 (δ_C_ 83.4), C-11 (δ_C_ 70.6), C-12 (δ_C_ 71.2), and C-17 (δ_C_ 72.0) were revealed by the HMBC correlations from H-3 (δ_H_ 5.31), H-11 (δ_H_ 5.47), H-12 (δ_H_ 5.38), and H-17 (δ_H_ 5.72) to the corresponding carbonyls of the acetoxyl groups, respectively. The remaining acetoxyl was subsequently assigned to C-2 on the basis of its downfield shifted carbon resonance at δ_C_ 81.1 (for the case of 2-OH, the C-2 carbon resonance normally appeared at *ca*. δ_C_ 78.0). The HMBC correlations between C-7 (δ_C_ 172.7) and H-6 (δ_H_ 2.31) and one of the oxygenated C-19 methylene signals at δ_H_ 5.00 (H-19a) indicated the presence of the characteristic C-6–C-7 appendage of a phragmalin-type limonoid and the six-membered C-7/C-19 δ-lactone ring. A HMBC correlation between H-15a (δ_H_ 2.62) and the ketal carbon resonance at δ_C_ 113.7 (C-31), instead of the correlation between H-15 and the C-16 carbonyl in common phragmalins, indicated that **1a** is a 16-decarboxylated phragmalin limonoid. The HMBC correlation between the ketal carbon and the methyl group signal H-32 (δ_H_ 1.65) suggested the linkage of the methyl to the ketal carbon, a biosynthetically extended C2 unit (C-31 and C-32) attached at C-15. The HMBC correlation between H-30 (δ_H_ 4.45) and the ketal carbon suggested the presence of an ether bridge between C-31 and C-30 (Figure 2). These data showed great similarity to those of chuktabularin B [10], except that a lactone carbonyl (δ_C_ 167.7) and a hemiacetal methine (δ_H_ 6.09; δ_C_ 95.7) signals replaced the corresponding two olefinic methine signals. HMBC correlations from H-17 to C-22, from H-22 and H-23 to C-21, and ^1^H-^1^H COSY correlation of H-22/H-23 indicated that a 23-hydroxy-20(22)-en-21,23-γ-lactone moiety instead of a β-furyl ring moiety located at C-17 in **1a**. The relative configuration of **1a** was elucidated using a ROESY experiment (Figure 3 and Table 1), in which the ROESY correlations of H-11/H-5, H-11/H-30, H-17/H-12, H-17/H-30 and 3-OAc/H-17, indicated that 3-OAc, H-5, H-11, H-12, H-17, and H-30 are co-facial and randomly assigned as β-oriented. ROESY correlations of Me-18/H-14, 9-OH/Me-18, 1-OH/Me-32, Me-32/2-OAc and H-29b/H-3 revealed that these protons adopt an α-orientation. The ROESY correlations of H-19a/1α-OH and H-19b/H-29a revealed that the six-membered 7,19-lactone ring was α-directed. Thus, the relative configuration of **1a** in solution was established by a ROESY experiment as depicted. Comparison of the NMR data of **1a** and **1b** indicated that they had a same planar core structure. The only significant differences between **1a** and **1b** were the chemical shifts of carbons around C-23 (Table 1), suggesting that stereochemistry at hemiacetal C-23 was to be epimerized. This tautomerism has also been found in similar compounds, such as dysoxylumic acid B [22] and walsogyne A [23], and compound **1** was named as chukbularisin A.

**Table 1:** NMR spectroscopic data of compound **1** (isomers **1a** and **1b**) in CDCl_3_ (δ in ppm, *J* in Hz).

No.	1a			1b		
	δ_C_^a^	δ_H_^b^	ROESY ^c^	δ_C_^a^	δ_H_^b^	ROESY ^c^
1	85.7 s			85.7 s		
2	81.1 s			81.1 s		
3	83.4 d	5.31 (s)	29b	83.3 d	5.31 (s)	29b
4	45.7 s			45.8 s		
5	40.8 d	2.00 (m)	11, 28	40.7 d	2.00 (m)	11, 28
6a	31.5 t	2.31 (m, 2H)		31.5 t	2.31 (m, 2H)	
6b						
7	172.7 s			172.8 s		
8	89.3 s			89.4 s		
9	75.0 s			75.2 s		
10	52.6 s			52.6 s		
11	70.6 d	5.47 (d, 3.8)	5, 12, 30	70.6 d	5.54 (d, 3.8)	5, 12, 30
12	71.2 d	5.38 (d, 3.8)	11, 17	71.4 d	5.38 (d, 3.8)	11, 17
13	41.8 s			41.8 s		
14	44.6 d	3.21 (dd, 12.2, 7.1)	18	44.5 d	3.21 (dd, 12.2, 7.1)	18
15a	35.4 t	2.62 (dd, 11.9, 7.1)		35.4 t	2.63 (dd, 11.9, 7.1)	
15b		1.94 (dd, 12.2, 11.9)			1.94 (dd, 12.2, 11.9)	
17	72.0 d	5.72 (s)	12, 30, 3-OAc	72.1 d	5.73 (s)	12, 30, 3-OAc
18	18.8 q	1.02 (s)	14, 9-OH	18.8 q	1.02 (s)	14, 9-OH
19a	69.5 t	5.00 (d, 12.5)	1-OH	69.5 t	4.97 (d, 12.5)	1-OH
19b		4.18 (dd, 12.5, 4.7)	29a		4.18 (dd, 12.5, 4.7)	29a
20	133.2 s			133.5 s		
21	167.7 s			167.7 s		
22	147.5 d	7.38 (br s)		148.3 d	7.34 (br s)	
23	95.7 d	6.09 (t, 10.9)		96.2 d	6.09 (t, 10.9)	
28	15.2 q	0.90 (s)	5	15.2 q	0.90 (s)	5
29a	38.9 t	2.09 (d, 11.8)	19b	38.9 t	2.09 (d, 11.8)	19b
29b		2.02 (d, 11.8)	3		2.02 (d, 11.8)	3
30	70.9 d	4.45 (s)	11, 17	70.9 d	4.42 (s)	11, 17
31	111.3 s			111.7 s		
32	18.8 q	1.65 (s)	1-OH, 2-OAc	18.8 q	1.65 (s)	1-OH, 2-OAc
2-OAc	170.2 s	2.09 (s)	32	170.1 s	2.09 (s)	32
	20.9 q			20.9 q		
3-OAc	168.7 s	2.45 (s)	17	168.9 s	2.45 (s)	17
	21.0 q			21.0 q		
11-OAc	171.5 s	2.13 (s)		171.2 s	2.12 (s)	
	20.8 q			20.8 q		
12-OAc	170.5 s	2.08 (s)		170.5 s	2.08 (s)	
	20.3 q			20.3 q		
17-OAc	170.7 s	2.11 (s)		170.7 s	2.11 (s)	
	20.2 q			20.2 q		
1-OH		4.86 (s)	32, 19a		4.85 (s)	32, 19a
9-OH		3.32 (s)	18		3.30 (s)	18

^a^ Recorded at 125 MHz; ^b^ Recorded at 500 MHz; ^c^ Recorded at 500 MHz.

Compound **2** was isolated as a white amorphous powder, and the IR absorbance bands at 3455, and 1745 cm^−1^, suggested the presence of hydroxyl and carbonyl groups. The molecular formula C_33_H_38_O_13_ was determined by the pseudomolecular ion peak at *m/z* 643.2383 ([M + H]^+^ calcd. 643.2385) in the HRESIMS, indicating 15 degrees of unsaturation. The ^13^C and DEPT NMR showed presence of six methyls, five methylenes, nine methines (three oxygenated, and three olefinic ones) and thirteen quaternary carbons (six oxygenated, and four ester carbonyls). The ^1^H- and ^13^C-NMR spectroscopic data were similar to those of andirolide V isolated from *Carapa guianensis* [24], except for the downfield-shifted C-10 carbon signal and the absence of the oxygenated C-19 methylene signals. Detailed analysis of the NMR data of **2** further revealed that the A, B, C, D, and E rings of a phragmalin-type limonoid remained intact. The isobutyryloxyl was assigned to C-30 (δ_C_ 69.8) by the HMBC correlations from H-30 (δ_H_ 5.64) to C-1’ of the isobutyryloxyl, while the only acetoxyl was attached to C-3 according to the HMBC correlation from H-3 (δ_H_ 4.83) to the acetoxyl carbonyl. The HMBC correlations from H-6a and H_2_-29 to the oxygenated and remarkably deshielded C-10 (δ_C_ 86.4) revealed the loss of CH_2_-19 and the formation of the five-membered 7,10-γ-lactone ring. The degrees of unsaturation of **2** and the 14 mass units less in its molecular formula compared to that of andirolide V further confirmed this deduction. Planar structure of **2** was finally characterized by analysis of ^1^H-^1^H COSY and HMBC data as depicted in Figure 2. The relative configuration of **2** was assigned the same as that of andirolide V based on the explanation of ROESY NMR analysis (Figure 3 and Table 2). Thus, compound **2** (chukbularisin B) was determined as a 19-norphragmalin limonoid, a rare pentanortriterpenoid that only two limonoids of this type had been reported to the best of our knowledge [14,15].

Compound **3** was obtained as a white amorphous powder. The molecular formula C_41_H_50_O_18_ was determined by the pseudomolecular ion peak at *m/z* 869.2623 ([M + K]^+^ calcd. 869.2629) in the HRESIMS. The IR spectrum of **3** exhibited absorptions for OH groups at 3464 cm^−1^ and an ester carbonyl at 1727 cm^−1^. The ^1^H- and ^13^C-NMR data of **3** (Table 2) showed highly similarity to those of chubularinsin H [21], except for the absence of NMR signals for an acetoxy group at C-6. Moreover, the chemical shift of C-6 (δ_C_ 33.1) in **3** was upfield shifted (*ca*. ∆δ_C_ 37.5 ppm) compared with that of chubularinsin H, indicating the lack of a 6-OAc. This inference was further supported by the 58 mass units less in its molecular formula compared to that of chubularinsin H and 2D NMR data. Finally, the planar structure of **3** was characterized by analysis of ^1^H-^1^H COSY and HMBC data as depicted in Figure 2.

The relative configuration of **3** was assigned the same as that of chubularinsin H based on the explanation of ROESY correlations (Figure 3 and Table 2). Thus, the structure of **3** (chukbularisin C) was determined to be a 6-deacetoxy derivative of chubularinsin H.

**Table 2:** NMR spectroscopic data of compounds **2** and **3** in CDCl_3_ (δ in ppm, *J* in Hz).

No.	2			3		
	δ_C_^a^	δ_H_^b^	ROESY ^c^	δ_C_^a^	δ_H_^b^	ROESY ^c^
1	84.8 s			83.0 s		
2	79.8 s			76.7 s		
3	82.8 d	4.83 (s)	28	85.9 d	5.49 (s)	29b
4	44.0 s			44.1 s		
5	39.1 d	2.94 (d, 8.4)	30	38.1 d	2.58 (d, 11.9)	12, 17, 28
6a	30.2 t	2.77 (d, 12.6)	29a	33.1 t	2.66 (d, 12.3)	
6b		2.59 (dd, 12.6, 8.4)			2.45 (d, 12.3	
7	174.3 s			173.9 s		
8	86.5 s			78.5 s		
9	84.5 s			90.6 s		
10	86.4 s			45.1 s		
11a	22.9 t	1.64 (overlapped)		75.0 d	4.17 (d, 3.6)	12, 19
11b		2.03 (m)				
12a	29.0 t	1.53 (m)		66.7 d	5.14 (br d, 3.6)	5, 11, 17
12b		1.41 (overlapped)				
13	34.9 s			31.3 s		
14	42.6 d	2.10 (dd, 10.6, 2.1)		31.1 s		
15a 15b	29.9 t	3.15 (dd, 19.6, 2.1)		69.4 d	7.16 (br d, 2.8)	17, 30
		2.72 (dd, 19.6, 10.6)				
16	169.8 s			167.1 s		
17	78.6 d	5.32 (s)	22, 30	70.2 d	6.42 (s)	5, 12, 15, 21
18a	20.3 q	1.15 (s)		18.8 t	2.64 (dd, 7.0, 3.1)	
18b					1.44 (d, 7.0)	
19				14.4 q	1.31 (s)	11, 29a
20	121.0 s			122.3 s		
21	141.0 d	7.47 (br s)		142.2 d	7.47 (br s)	17
22	109.7 d	6.40 (br s)	17	109.9 d	6.50 (br d, 1.6)	
23	143.6 d	7.43 (br s)		143.4 d	7.39 (br t, 1.6)	
28	14.3 q	1.01 (s)	3	14.8 q	0.83 (s)	5, 29b
29a	39.5 t	1.87 (s, 2H)	6b	39.0 t	1.92 (s, 2H)	19
29b						3, 28
30	69.8 d	5.64 (s)	5, 17	69.4 d	5.39 (s)	15, 3-OAc
31	119.8 s			119.9 s	1.66 (s)	3′
32	21.0 q	1.75 (s)		16.4 q		
3-OAc	170.1 s	2.19 (s)		169.3 s	2.22 s	30
	21.6 q			21.2 q		
12-OAc				170.9 s	1.66 (s)	
				20.0 q		
7-OCH_3_				52.6 q	3.75 (s)	
15-OCOCHMe_2_						
1’				177.9 s		
2’				34.2 d	2.92 (m)	
3’				19.9 q	1.32 (d, 7.0)	
4’				18.0 q	1.25 (d, 7.0)	
30-OCOCHMe_2_						
1’	175.4 s			173.9 s		
2’	34.6 d	2.56-2.61 (m)		34.0 d	2.51 (m)	
3’	18.2 q	1.11 (d, 7.0)		19.5 q	1.19 (d, 7.0)	32
4’	19.3 q	1.19 (d, 7.0)		18.9 q	1.17 (d, 7.0)	
1-OH					2.85 (s)	
2-OH		2.85 (s)			3.38 (s)	

^a^ Recorded at 125 MHz; ^b^ Recorded at 500 MHz; ^c^ Recorded at 500 MHz.

Compound **4** was isolated as a white amorphous powder and the IR absorbance bands at 3454 and 1735 cm^−1^ suggested the presence of hydroxyl and carbonyl groups. The molecular formula C_41_H_48_O_20_ was determined by the pseudomolecular ion peak at 883.2627 *m/z* ([M + Na]^+^ calcd. 883.2631) in the HRESIMS, indicating 18 degrees of unsaturation. The ^13^C and DEPT NMR showed the presence of ten methyls, two methylenes, twelve methines and seventeen quaternary carbons. The combined features of its ^1^H- and ^13^C-NMR spectra suggested that compound **4** was also a phragmalin-type limonoid with a β-substituted furanyl ring and typical CH_2_-29 proton signals of 4,29 1-ring-bridge in phragmalins. Furthermore, comparison of the ^1^H- and ^13^C-NMR data (Table 3) of **4** with those of tabularisin R [25] indicated that their structures showed high similarity. The only structural difference between them was in the presence of one additional acetoxyl group at C-3 in **4** replacing the 3-OH in tabularisin R, which was further confirmed by the downfield shifted H-3 (∆δ_H_ 1.55 ppm) signal of **4** owning to the acetylation effect, and the HMBC correlation from H-3 (δ_H_ 5.36) to the carbonyl (δ_C_ 169.0). The relative configuration of **4** was assigned the same as that of tabularisin R based on the explanation of ROESY correlations (Figure 3 and Table 3). Thus, the structure of **4** (chukbularisin D) was determined to be a 3-*O-*acetyl derivative of tabularisin R.

Compound **5** was isolated as a white amorphous powder. The molecular formula C_43_H_50_O_21_ was determined by the pseudomolecular ion peak at *m/z* 925.2737 ([M + Na]^+^ calcd. 925.2737) in the HRESIMS. IR data exhibited the presence of hydroxyls (3452 cm^−1^) and carbonyl groups (1736 cm^−1^). Comparison of the ^1^H- and ^13^C-NMR data (Table 3) of **5** with those of tabularisin C [7] indicated that their structures were closely related, and that they only differed in the nature of the oxygenated group at C-11. The corresponding HMBC correlation between the acetoxyl carbonyl and H-11 (δ_H_ 5.61) indicated that the 11-OH in tabularisin C was replaced by a 11-OAc group in **5**. Finally, the planar structure of **5** was characterized by analysis of ^1^H-^1^H COSY and HMBC data as depicted in Figure 2. The relative configuration of **5** was established to be the same as tabularisin C by the ROESY data (Figure 3 and Table 3). Thus, the structure of **5** was elucidated and it was named chukbularisin E.

**Table 3:** NMR spectroscopic data of compounds **4** and **5** in CDCl_3_ (δ in ppm, *J* in Hz).

No.	4			5		
	δ_C_^a^	δ_H_^b^	ROESY ^c^	δ_C_^a^	δ_H_^b^	ROESY ^c^
1	84.6 s			83.9 s		
2	76.0 s			83.1 s		
3	85.5 d	5.36 (s)	29b	85.8 d	5.27 (s)	
4	44.4 s			44.6 s		
5	44.7 d	2.81 (br s)	12, 17, 28, 30	43.9 d	2.80 (s)	12, 17, 28, 30
6	71.3 d	6.26 (br s)	19	71.2 d	6.22 (s)	19
7	172.1 s			172.1 s		
8	86.6 s			86.5 s		
9	84.2 s			84.7 s		
10	49.4 s			49.5 s		
11	67.1 d	5.66 (d, 4.9)	12, 15, 19	67.0 d	5.61 (d, 4.9)	12, 15, 19
12	66.7 d	5.42 (d, 4.9)	5, 11, 17	66.5 d	5.47 (d, 4.9)	5, 11, 17
13	29.6 s			29.8 s		
14	25.1 s			24.9 s		
15	69.7 d	6.94 (br s)	11, 17, 30	70.5 d	6.99 (d, 2.6)	11, 17, 30
16	166.0 s			165.7 s		
17	72.1 d	6.50 (s)	5, 12, 15, 21	71.8 d	6.44 (s)	5, 12, 15, 21
18a	16.2 t	2.70 (dd, 7.2, 2.5)	22	17.8 t	2.71 (dd, 7.2, 2.6)	
18b		1.51 (d, 7.2)			1.43 (br d, 7.2)	
19	17.8 q	1.37 (s)	6, 11, 29a	17.6 q	1.36 (s)	6, 11, 29a
20	122.2 s			122.2 s		
21	142.1 d	7.49 (br s)	17	142.1 d	7.52 (br s)	17
22	109.7 d	6.49 (br d, 1.4)	18b	109.9 d	6.51 (br d, 1.3)	
23	143.4 d	7.38 (br t, 1.6)		143.4 d	7.38 (br t, 1.7)	
28	15.6 q	0.96 (s)	5, 29b	15.6 q	0.92 (s)	5, 29b
29a	40.2 t	2.16 (d, 11.0)	19	40.8 t	1.71 (br d, 11.4)	19
29b		1.83 (d, 11.0)	3, 28		2.28 (br d, 11.4)	28
30	79.4 d	4.09 (s)	5, 15, 3-OAc	76.0 d	5.05 (s)	5, 15
31	119.4 s			116.2 s		
32	29.2 d	2.15 (m)		15.8 q	1.66 (s)	
33	17.1 q	1.07 (d, 7.0)				
34	17.0 q	1.05 (d, 7.0)				
3-OAc	169.0 s	2.21 (s)	30	168.6 s	2.33 (s)	
	21.1 q			20.6 q		
6-OAc	169.3 s	2.21 (s)		168.9 s	2.20 (s)	
	21.3 q			21.1 q		
11-OAc	169.2 s	2.05 (s)		169.0 s	2.07 (s)	
	20.9 q			21.2 q		
12-OAc	170.1 s	1.53 (s)		170.1 s	1.54 (s)	
	19.3 q			19.3 q		
15-OAc	169.0 s	2.23 (s)		169.2 s	2.22 (s)	
	21.1 q			20.9 q		
7-OCH_3_	53.7 q	3.79 (s)		53.7 q	3.79 (s)	
2-OCOCHMe_2_						
1’				175.9 s		
2’				34.6 d	2.50-2.55 (m)	
3’				18.9 q	1.17 (d, 7.0)	
4’				18.9 q	1.20 (d, 7.0)	
1-OH		3.28 (s)			3.50 (s)	
2-OH		3.47 (s)				

^a^ Recorded at 125 MHz; ^b^ Recorded at 500 MHz; ^c^ Recorded at 500 MHz.

The known compounds were identified as tabularisin E (**6**) [26], chubularisin E (**7**) [21], chubularisin K (**8**) [21], chukvelutilide B (**9**) [9], chukvelutilide D (**10**) [9], and chukvelutilide H (**11**) [25], respectively, by interpreting their data and making comparisons with literature values.

α-Glucosidase inhibitors are used in the treatment of non-insulin-dependent diabetes mellitus. In order to find *in vitro* α-glucosidase inhibitory agents among these compounds, some optimizations had been done to the reaction system, which was referred to Li [27]. The results showed that compounds **2**, **3**, **4**, **5**, and **8** exhibited α-glucosidase inhibitory activity with IC_50_ values of 0.06 ± 0.008, 0.04 ± 0.002, 0.52 ± 0.039, 1.09 ± 0.040, and 0.20 ± 0.057 mM, respectively (Table 4), among which compound **3** is 24 times more potent than the positive control (acarbose, IC_50_ 0.95 ± 0.092 mM). Structure–activity relationship analysis revealed that the furanyl ring and the C-16/17 δ-lactone ring in these phragmalin limonoids are important for the α-glucosidase inhibitory activity. Thus, phragmalin limonoids might be promising agents for treatment and prevention of diabetes and need be further investigated for this purpose.

**Table 4:** *In vitro* α-glucosidase inhibitory activities of compounds **1**–**11**.

Compound	IC_50_ Value (mM) ^a^	Compound	IC_50_ Value (mM) ^a^
**1**	–	**7**	–
**2**	0.06 ± 0.008	**8**	0.20 ± 0.057
**3**	0.04 ± 0.002	**9**	–
**4**	0.52 ± 0.039	**10**	–
**5**	1.09 ± 0.040	**11**	–
**6**	–	Acarbose ^b^	0.95 ± 0.092

^a^ Values present mean ± SD of triplicate experiments; ^b^ Positive control; “–”inactive.

## 3. Experimental Section

### 3.1. General Procedures

Optical rotations were measured on an Autopol III polarimeter (Rudolph Research Analytical, Hackettstown, NJ, USA). Melting points were determined on a Beijing Taike X-5 stage apparatus (Beijing Taike Instrument Company, Beijing China) and are uncorrected. UV spectra were recorded on a DU800 spectrophotometer (Beckman, Brea, CA, USA). IR spectra were obtained on a 380 FT-IR spectrometer (Thermo, Pittsburgh, PA, USA). NMR experiments were recorded for ^1^H-NMR at 500 MHz and ^13^C-NMR at 125 MHz on an AV III spectrometer (Bruker, Bremen, Germany) using TMS as an internal standard. HRESIMS were acquired using an API QSTAR Pulsar mass spectrometer (Bruker). Column chromatographic separations were carried out by using silica gel (60–80 mesh and 200–300 mesh; Qingdao Haiyang Chemical Group Corporation, Qingdao, China), MCI gel CHP-20P (75–150 μm; Mitsubishi Chemical Industries Co. Ltd., Tokyo, Japan), Rp-18 (20–45 μm; Fuji Silysia Chemical Ltd., Durham, NC, USA) and Sephadex LH-20 (40–70 μm; Merck, Darmstadt, Germany). Silica gel (200–300 mesh), silica gel H (10–40 μm) and precoated silica GF_254_ plates for analytical TLC were produced by Qingdao Haiyang Chemical Company, Ltd. The spots on TLC were visualized by spraying with 5% H_2_SO_4_-ethanol solution.

### 3.2. Plant Material

The stems of *Chukrasia tabularis* were collected in Haikou, Hainan Province, P.R. China, in July 2014, which was identified by Dr. Jun Wang, Institute of Tropical Bioscience and Biotechnology, Chinese Academy of Tropical Agriculture Science, where a voucher specimen (No. 20140726) was deposited.

### 3.3. Extraction and Isolation

The air-dried stems of *C. tabularis* (110.0 kg) were pulverized and extracted with 95% ethanol (314 L) three times (7, 5, 3 days), at room temperature. The combined ethanol extract was then filtered through absorbent gauze, and the filtrate was concentrated under reduced pressure to remove the ethanol. Then, the residue (13.7 kg) was suspended in H_2_O and partitioned with petroleum ether, EtOAc, and *n*-BuOH successively. All the extracts were separately combined and evaporated to dryness under reduced pressure. These three fractions were designated as PEF (30.0 g), EAF (1700.0 g), and BUF (800.0 g), respectively. According to TLC analysis, the EtOAc fraction (1700.0 g) was separated into 18 fractions on a silica gel column (30 × 120 cm) using a step gradient elution of petroleum ether–EtOAc (20:1, 10:1, 5:1, 2:1, 1:1, and 0:1, *v*/*v*). Fr.17 (120.0 g) was subjected to silica gel (10 × 55 cm) vacuum liquid chromatography and eluted with CHCl_3_–MeOH (1:0, 100:1, 50:1, 25:1, 15:1, 10:1, 5:1, 2:1, 1:1, and 0:1, *v*/*v*) to provide 10 fractions (Fr.17-1–Fr.17-10). Fr.17-1 (3.5 g) was applied to ODS gel (3 × 40 cm) eluting with MeOH–H_2_O (from 3:7 to 1:0) to yield Fr.17-1-1–7. Fr.17-1-5 (350.0 mg) was chromatographed on Sephadex LH-20 gel (3 × 100 cm) with CHCl_3_–MeOH (*v*/*v*, 1:1), followed by silica gel (1.2 × 50 cm) eluting with petroleum ether–EtOAc (*v*/*v*, 6:4) to afford compound **1** (4.0 mg). Fr.15 (268.0 g) was subjected to silica gel (10 × 55 cm) vacuum liquid chromatography and eluted with CHCl_3_–EtOAc (1:0, 20:1, 10:1, 5:1, 1:1, and 0:1, *v*/*v*) to provide eight fractions (Fr.15-1–Fr.15-8). Fr.15-2 (36.8 g) was first subjected to a MCI gel column, eluted with MeOH–H_2_O (from 5:5 to 1:0) to yield Fr.15-2-1–15-2-4. Fr.15-2-1 (9.0 g) was applied to ODS gel (3 × 40 cm) eluting with MeOH–H_2_O (from 3:7 to 1:0) to yield Fr.15-2-1-1–20. Fr.15-2-1-5 (220.0 mg) was chromatographed on Sephadex LH-20 gel (3 × 100 cm) with CHCl_3_–MeOH (*v*/*v*, 1:1), followed by silica gel (1.2 × 50 cm) eluting with petroleum ether–EtOAc (*v*/*v*, 8:3) to afford **2** (8.0 mg) and **11** (8.0 mg). Fr.15-2-1-11 (850.0 mg) was chromatographed on Sephadex LH-20 gel (3 × 100 cm) with CHCl_3_–MeOH (*v*/*v*, 1:1), followed by silica gel (1.2 × 50 cm) eluting with petroleum ether–CHCl_3_–isopropanol (*v*/*v*/*v*, 5:5:0.07) to afford **3** (3.5 mg) and **8** (10.0 mg). Fr.15-2-1-13 (580.0 mg) was chromatographed on Sephadex LH-20 gel (3 × 100 cm) with CHCl_3_–MeOH (*v*/*v*, 1:1), followed by silica gel (1.2 × 50 cm) eluting with petroleum ether–EtOAc (*v*/*v*, 10:3) to afford compound **9** (15.0 mg) and **10** (8.0 mg). Fr.15-3 (26.8 g) was first subjected to a MCI gel column, eluted with MeOH–H_2_O (from 5:5 to 1:0) to yield Fr.15-3-1–15-3-8. Fr.15-3-3 (5.0 g) was applied to ODS gel (3 × 40 cm) eluting with MeOH–H_2_O (from 3:7 to 1:0) to yield Fr.15-3-3-1–18. Fr.15-3-3-5 (250.0 mg) was chromatographed on Sephadex LH-20 gel (3 × 100 cm) with CHCl_3_–MeOH (*v*/*v*, 1:1), followed by silica gel (1.2 × 50 cm) eluting with petroleum ether–CHCl_3_–isopropanol (*v*/*v*/*v*, 5:5:0.06) to afford **4** (7.0 mg) and **6** (10 mg). Fr.15-3-3-10 (300.0 mg) was chromatographed on Sephadex LH-20 gel (3 × 100 cm) with CHCl_3_–MeOH (*v*/*v*, 1:1), followed by silica gel (1.2 × 50 cm) eluting with petroleum ether–EtOAc (*v*/*v*, 8:3) to afford **5** (4.6 mg) and **7** (10 mg).

*Chukbularisin A* (**1**): White amorphous powder; mp 201–203 °C; ${\left[\alpha \right]}_{D}^{28}$ = +55° (*c* 0.30, CHCl_3_); UV (CHCl_3_): λ_max_ (log ε) 240 (3.60) nm; IR (KBr) *ν*_max_ 3443, 2923, 2853, 1746, 1636, 1217, 1043, 598 cm^−1^; ^1^H- and ^13^C-NMR data see Table 1; positive-mode HRESIMS *m*/*z* 810.2817 [M + NH_4_]^+^ (calcd. for C_37_H_44_O_19_NH_4_, 810.2815).

*Chukbularisin B* (**2**): White amorphous powder; mp 185–186 °C; ${\left[\alpha \right]}_{D}^{28}$ = +123° (*c* 0.20, CHCl_3_); UV (CHCl_3_) λ_max_ (log ε): 240 (3.37) nm; IR (KBr) *ν*_max_ 3455, 2923, 1745, 1640, 1215, 1072, 760 cm^−1^; ^1^H- and ^13^C-NMR data see Table 1; positive-mode HRESIMS *m*/*z* 643.2383 [M + H]^+^ (calcd. for C_33_H_39_O_13_, 643.2385).

*Chukbularisin C* (**3**): White amorphous powder; mp 198–199 °C; ${\left[\alpha \right]}_{D}^{28}$ = +68° (*c* 0.20, CHCl_3_); UV (CHCl_3_): λ_max_ (log ε) 240 (3.61) nm; IR (KBr) *ν*_max_ 3464, 2954, 1727, 1655, 1278, 1119, 1074 cm^−1^; ^1^H- and ^13^C-NMR data see Table 2; positive-mode HRESIMS *m*/*z* 869.2623 [M + K]^+^ (calcd. for C_41_H_50_O_18_K, 869.2629).

*Chukbularisin D* (**4**): White amorphous powder; mp 190–191 °C; ${\left[\alpha \right]}_{D}^{28}$ = +146° (*c* 0.05, CHCl_3_); UV (CHCl_3_): λ_max_ (log ε) 248 (4.35) nm; IR (KBr) *ν*_max_ 3454, 2926, 2088, 1735, 1634, 1383, 503 cm^−1^; ^1^H- and ^13^C-NMR data see Table 2; positive-mode HRESIMS *m*/*z* 883.2627 [M + Na]^+^ (calcd. for C_41_H_48_O_20_Na, 883.2631).

*Chukbularisin E* (**5**): White amorphous powder; mp 208–209 °C; ${\left[\alpha \right]}_{D}^{28}$ = +135° (*c* 0.10, CHCl_3_); UV (CHCl_3_): λ_max_ (log ε) 246 (3.87) nm; IR (KBr) *ν*_max_ 3452, 2923, 1736, 1638, 1383, 1099, 491 cm^−1^; ^1^H- and ^13^C-NMR data see Table 2; positive-mode HRESIMS *m*/*z* 925.2737 [M + Na]^+^ (calcd. for C_43_H_50_O_21_Na, 925.2737). (See Figures S1–S40 for more details about the original spectra of NMR and positive-mode HRESIMS data for the compounds **1**–**5**).

### 3.4. α-Glucosidase Inhibitory Assays

The compounds tested *in vitro* for α-glucosidase activities were performed on the UV spectrophotometer, and the method used was that of Li [27]. The optimized procedure was as follows: 20 μL of 0.2 U/mL α-glucosidase has been added into 0.1mM potassium phosphate buffer (pH 6.8, 112 μL), then mixed with the testing sample (8 μL). After being preincubated at 37 °C for 15 min, 20 μL of 2.5 mmol/L 4-nitrophenyl-α-d-glucopyranoside was added and then mixed. The reaction was carried out at 37 °C for 15 min and stopped by adding 0.2 M solution of Na_2_CO_3_ (80 μL). The optical density values of the reaction mixture were the mean values of three measurements, which were performed at 405 nm wavelength. Acarbose (National Institutes for Food and Drug Control, Beijing, China, purity > 99.99%) was used as the positive control.

## 4. Conclusions

In conclusion, eleven limonoids including five new ones were isolated from the stems of *C. tabularis* based on its α-glucosidase inhibitory activity. Compounds **2**, **3**, **4**, **5**, and **8** displayed comparable or stronger α-glucosidase inhibition activity than acarbose (IC_50_ 0.95 ± 0.092 mM) with IC_50_ values of 0.06 ± 0.008, 0.04 ± 0.002, 0.52 ± 0.039, 1.09 ± 0.040, and 0.20 ± 0.057 mM, respectively. It is worth noting that compound **3** is 24 times more potent than acarbose, and may serve as an attractive leading compound for the development of potent α-glucosidase inhibition agents.

## Supplementary Materials

Supplementary materials can be accessed at: http://www.mdpi.com/1420-3049/21/1/58/s1.
