# Supplementary material for: New Phragmalin-Type Limonoids from Chukrasia tabularis and Their α-Glucosidase Inhibitory Activity
Source: Molecules. 2016 Jan 5;21(1):58. doi: 10.3390/molecules21010058 (PMC6274087; doi:10.3390/molecules21010058)
Supplement: Supplementary file 1 [file molecules-21-00058-s001.pdf]

Supplementary Materials: New Phragmalin-Type Limonoids from *Chukrasia tabularis* and Their *α*-Glucosidase Inhibitory Activity

Jun-Lin Peng, Jun Wang, Fan-Dong Kong, Zi-Qi Liu, Pei Wang, Bei Jiang, Cui-Juan Gai, Wen-Li Mei and Hao-Fu Dai

Contents

Figure S1. ^{1}H-NMR (500 MHz) spectrum of the new compound **1** in $CDCl_3$.

Figure S2. ^{13}C-NMR (125 MHz) and DEPT NMR spectrum of the new compound **1** in $CDCl_3$.

Figure S3. HSQC spectrum of the new compound **1** in $CDCl_3$.

Figure S4. ^{1}H-^{1}H COSY spectrum of the new compound **1** in $CDCl_3$.

Figure S5. HMBC spectrum of the new compound **1** in $CDCl_3$.

Figure S6. ROESY spectrum of the new compound **1** in $CDCl_3$.

Figure S7. Partial enlarged ROESY spectra of compound **1** in $CDCl_3$.

Figure S8. ^{1}H -NMR (500 MHz) spectrum of the new compound **2** in $CDCl_3$.

Figure S9. ^{13}C-NMR (125 MHz) and DEPT NMR spectrum of the new compound **2** in $CDCl_3$.

Figure S10. HSQC spectrum of the new compound **2** in $CDCl_3$.

Figure S11. ^{1}H-^{1}H COSY spectrum of the new compound **2** in $CDCl_3$.

Figure S12. HMBC spectrum of the new compound **2** in $CDCl_3$.

Figure S13. ROESY spectrum of the new compound **2** in $CDCl_3$.

Figure S14. Partial enlarged ROESY spectra of compound **2** in $CDCl_3$.

Figure S15. ^{1}H-NMR (500 MHz) spectrum of the new compound **3** in $CDCl_3$.

Figure S16. ^{13}C-NMR (125 MHz) and DEPT NMR spectrum of the new compound **3** in $CDCl_3$.

Figure S17. HSQC spectrum of the new compound **3** in $CDCl_3$.

Figure S18. ^{1}H-^{1}H COSY spectrum of the new compound **3** in $CDCl_3$.

Figure S18. HMBC spectrum of the new compound **3** in $CDCl_3$.

Figure S20. ROESY spectrum of the new compound **3** in $CDCl_3$.

Figure S21. Partial enlarged ROESY spectra of compound **3** in $CDCl_3$.

Figure S22. ^{1}H-NMR (500 MHz) spectrum of the new compound **4** in $CDCl_3$.

Figure S23. ^{13}C-NMR (125 MHz) and DEPT NMR spectrum of the new compound **4** in $CDCl_3$.

Figure S24. HSQC spectrum of the new compound **4** in $CDCl_3$.

Figure S25. ^{1}H-^{1}H COSY spectrum of the new compound **4** in $CDCl_3$.

Figure S26. HMBC spectrum of the new compound **4** in $CDCl_3$.

Figure S27. ROESY spectrum of the new compound **4** in $CDCl_3$.

Figure S28. Partial enlarged ROESY spectra of compound **4** in $CDCl_3$.

Figure S29. ^{1}H-NMR (500 MHz) spectrum of the new compound **5** in $CDCl_3$.

Figure S30. ^{13}C-NMR (125 MHz) and DEPT NMR spectrum of the new compound **5** in $CDCl_3$.

Figure S31. HSQC spectrum of the new compound **5** in $CDCl_3$.

Figure S32. ^{1}H-^{1}H COSY spectrum of the new compound **5** in $CDCl_3$.

Figure S33. HMBC spectrum of the new compound **5** in $CDCl_3$.

Figure S34. ROESY spectrum of the new compound **5** in $CDCl_3$.

Figure S35. Partial enlarged ROESY spectra of compound **5** in $CDCl_3$.

Figure S36. HR-ESI-MS spectrum of the new compound **1**.

Figure S37. HR-ESI-MS spectrum of the new compound **2**.

Figure S38. HR-ESI-MS spectrum of the new compound **3**.

Figure S39. HR-ESI-MS spectrum of the new compound **4**.

Figure S40. HR-ESI-MS spectrum of the new compound **5**.

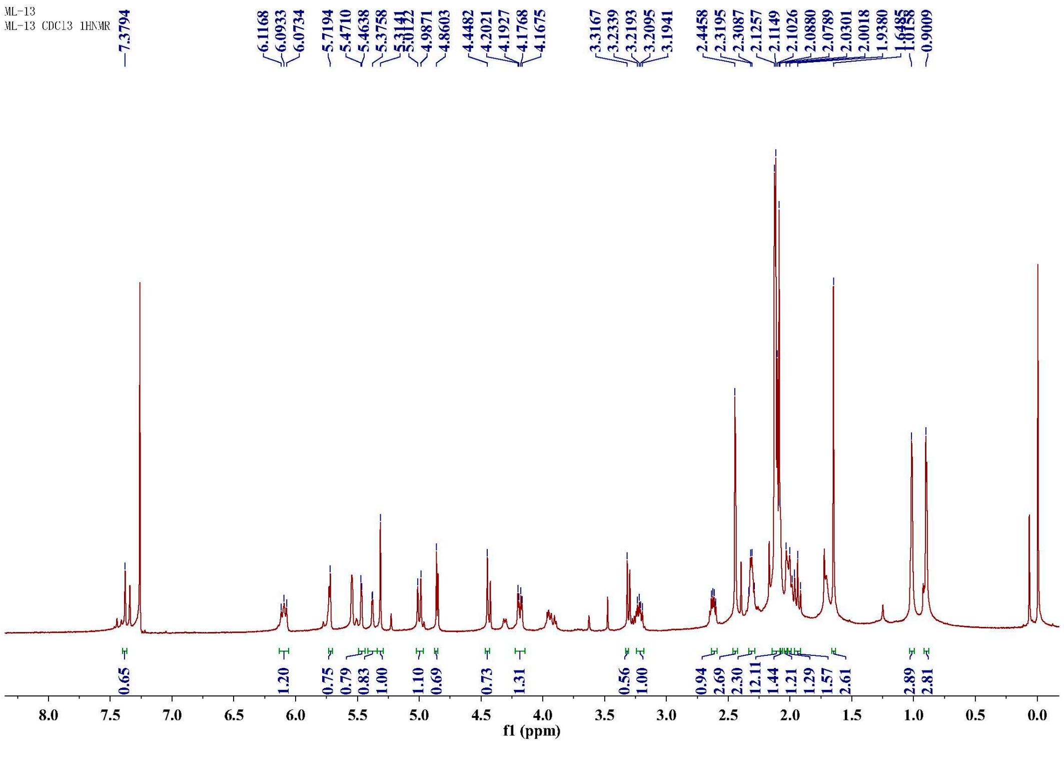

Figure S1. ^{1}H-NMR (500 MHz) spectrum of the new compound **1** in $CDCl_3$.

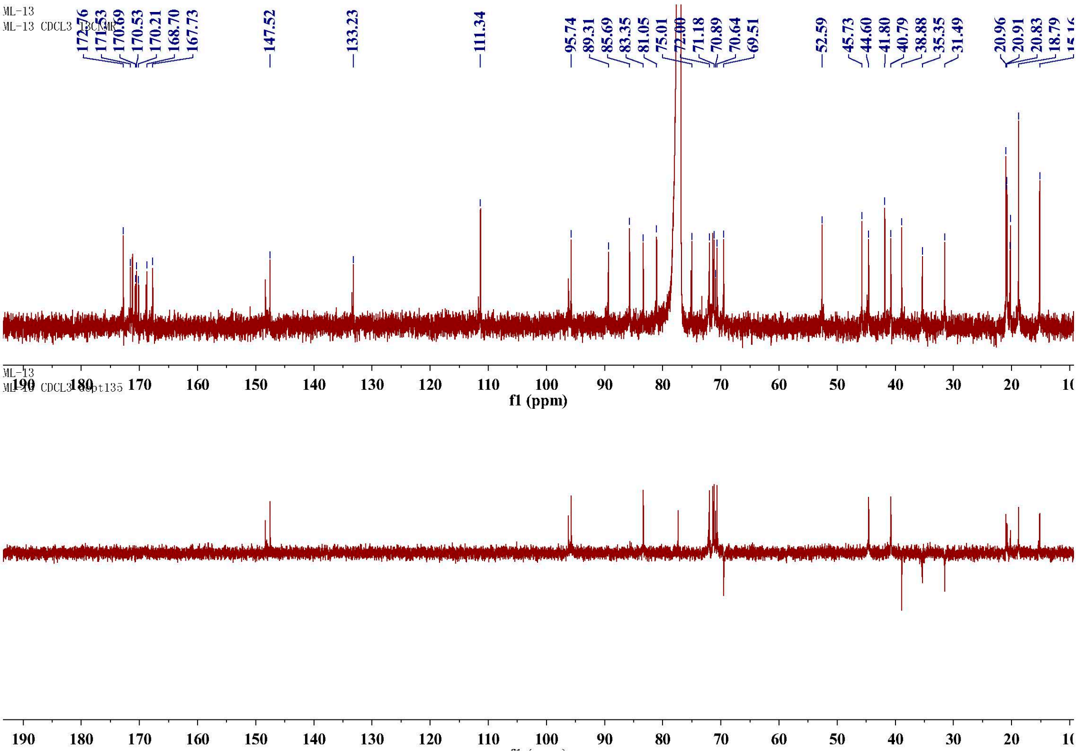

Figure S2. ^{13}C-NMR (125 MHz) and DEPT NMR spectrum of the new compound **1** in $CDCl_3$.

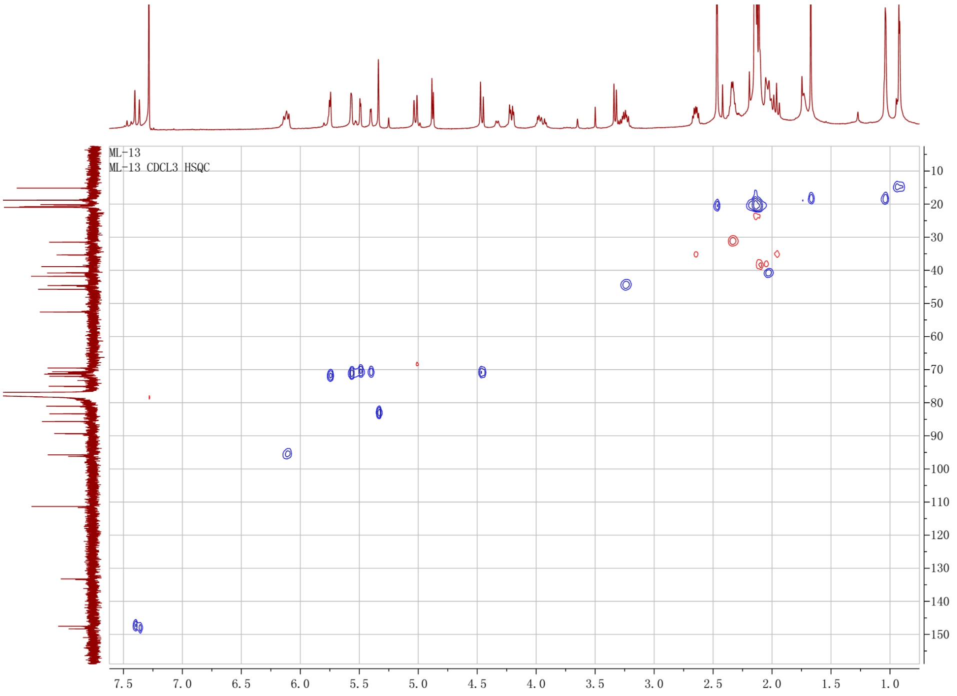

Figure S3. HSQC spectrum of the new compound **1** in $CDCl_3$.

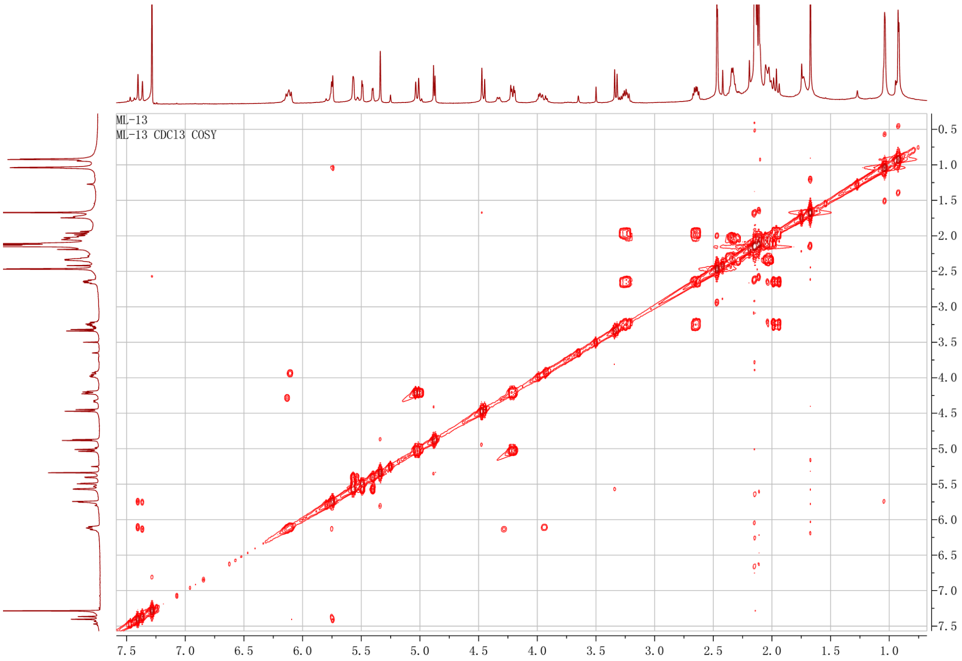

Figure S4. ^1H-^1H COSY spectrum of the new compound **1** in $CDCl_3$.

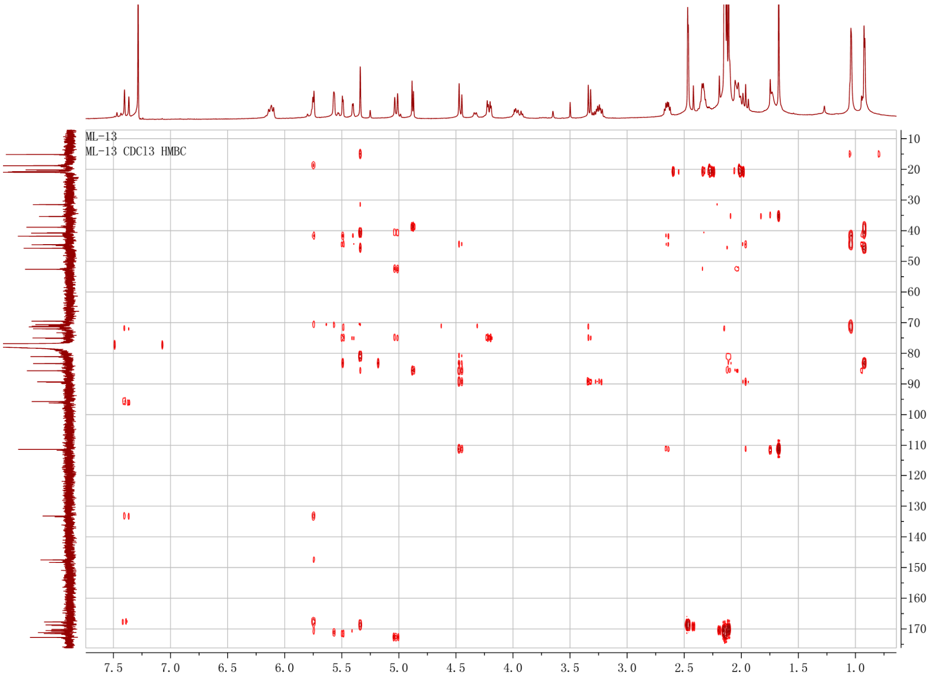

Figure S5. HMBC spectrum of the new compound **1** in $CDCl_3$.

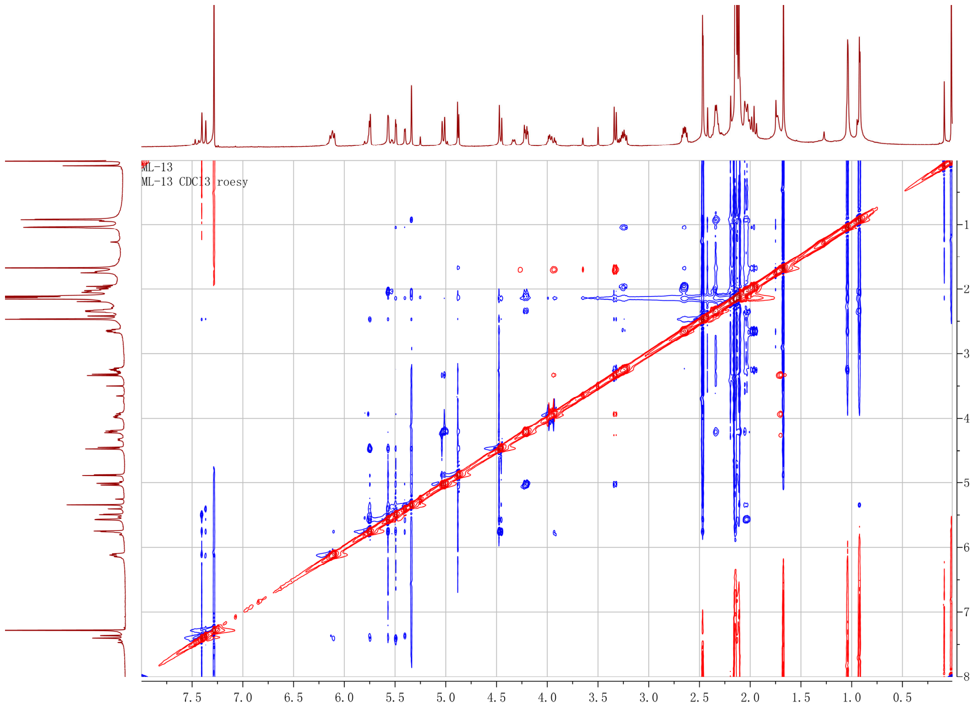

Figure S6. ROESY spectrum of the new compound **1** in $CDCl_3$.

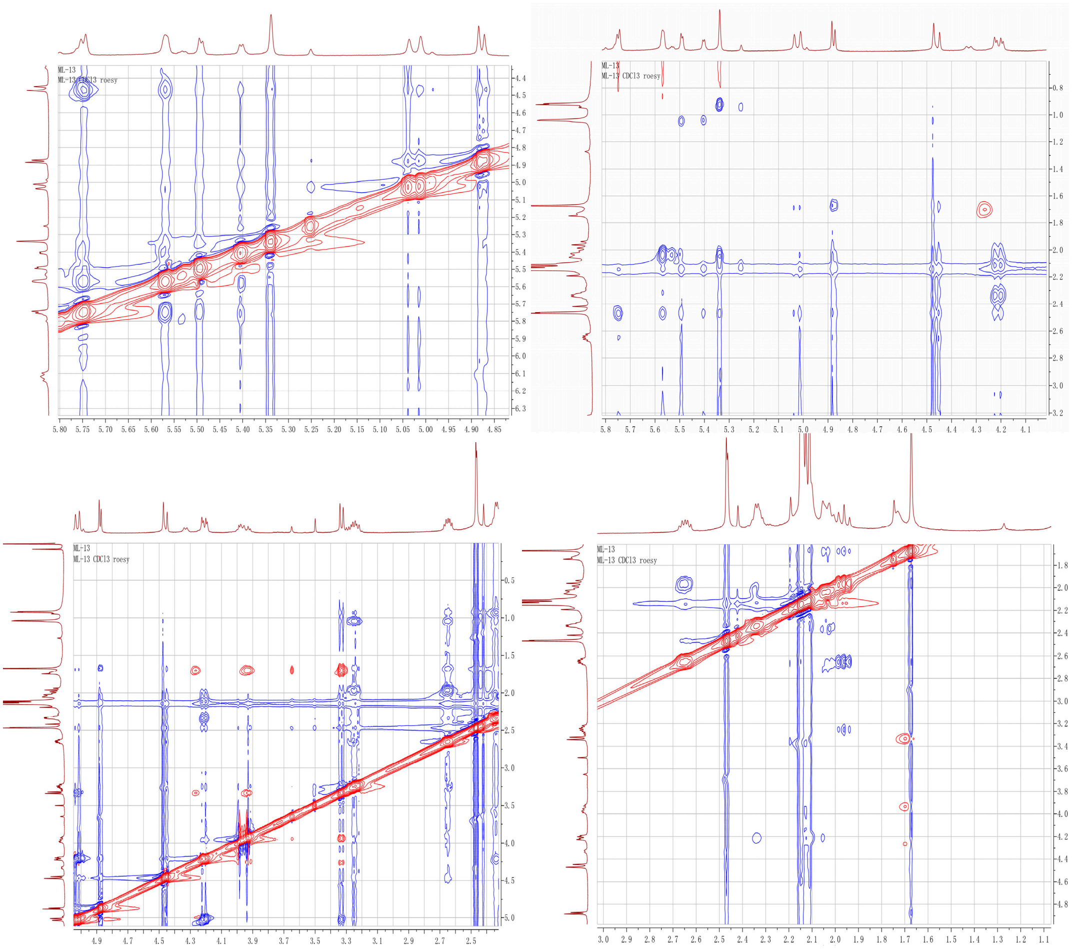

Figure S7. *Cont.*

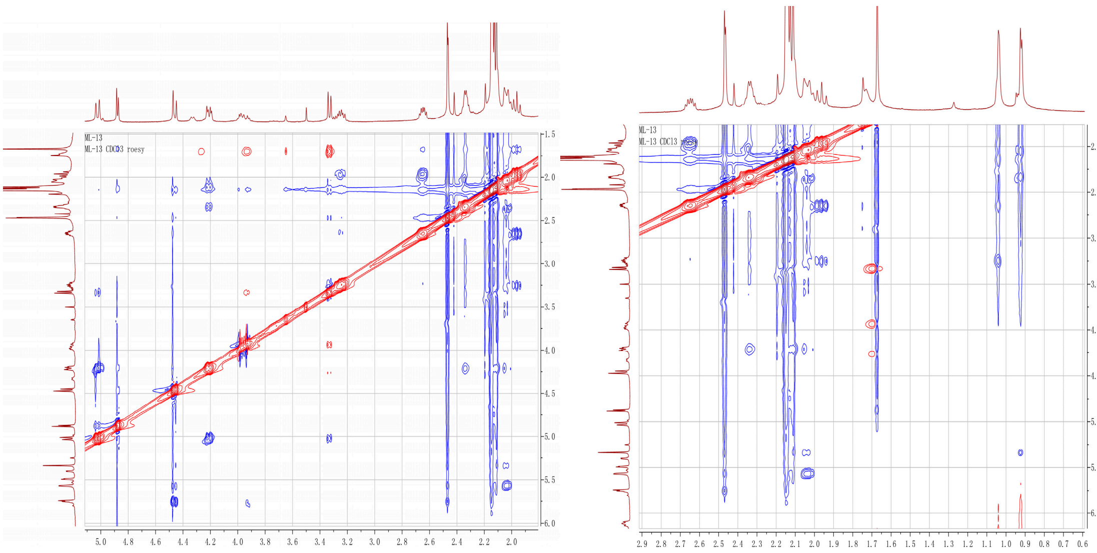

Figure S7. Partial enlarged ROESY spectra of compound **1** in CDCl3.

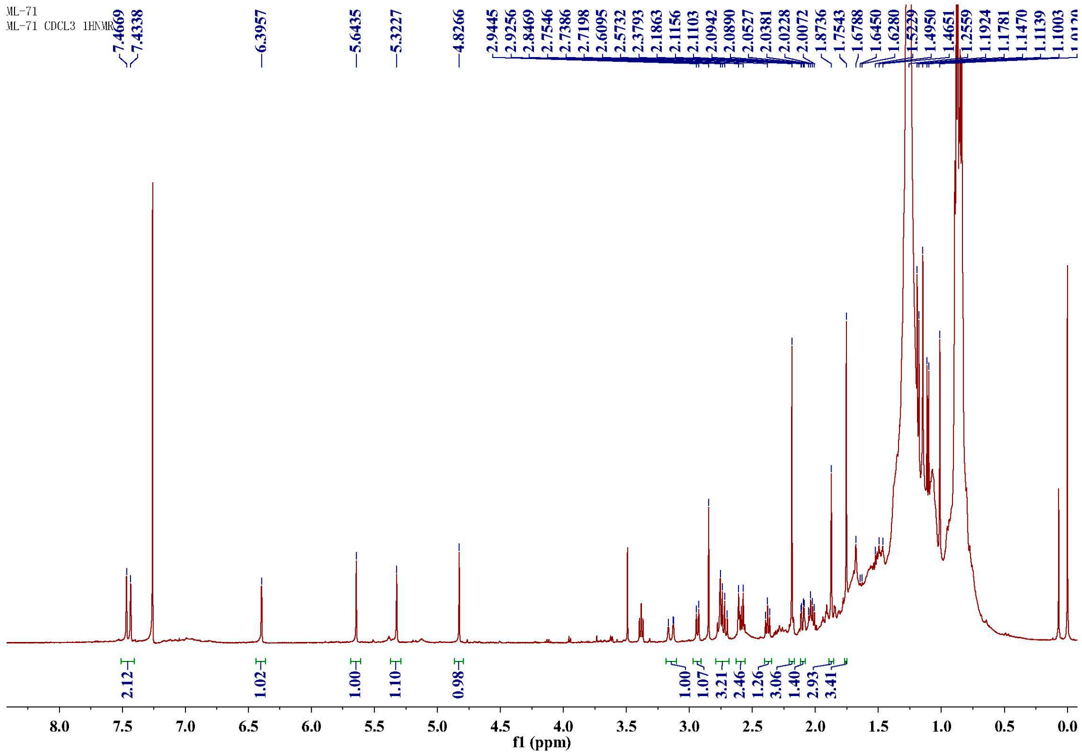

Figure S8. ^{1}H-NMR (500 MHz) spectrum of the new compound **2** in CDCl3.

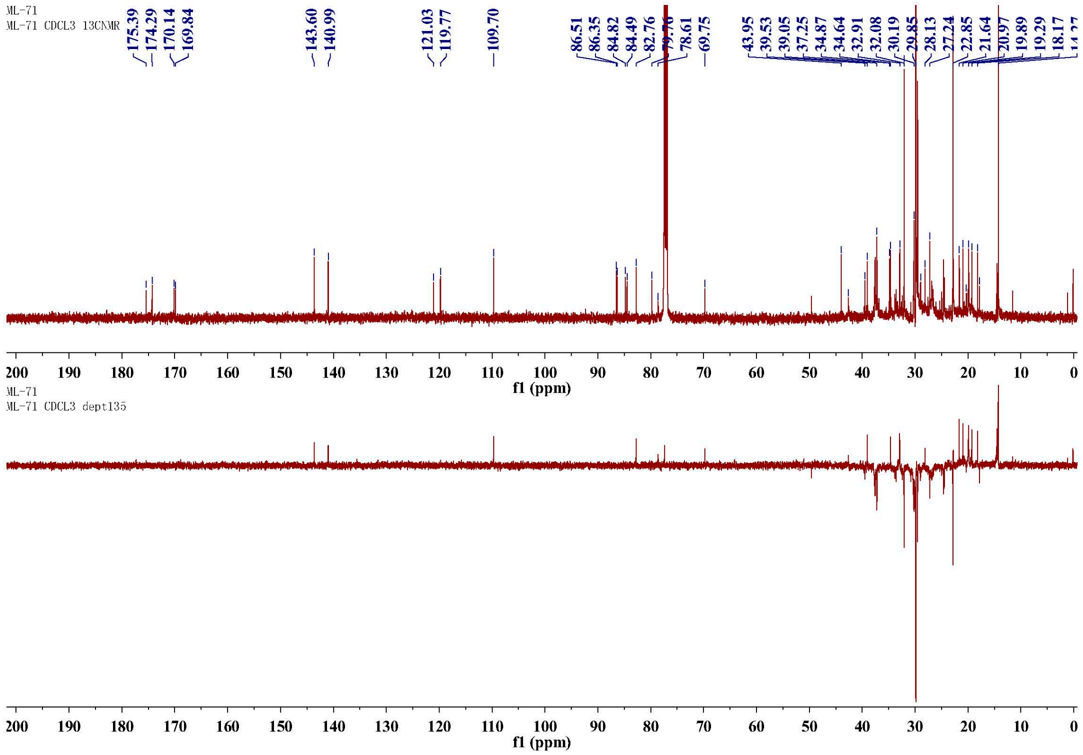

Figure S9. ^{13}C-NMR (125 MHz) and DEPT NMR spectrum of the new compound **2** in $CDCl_3$.

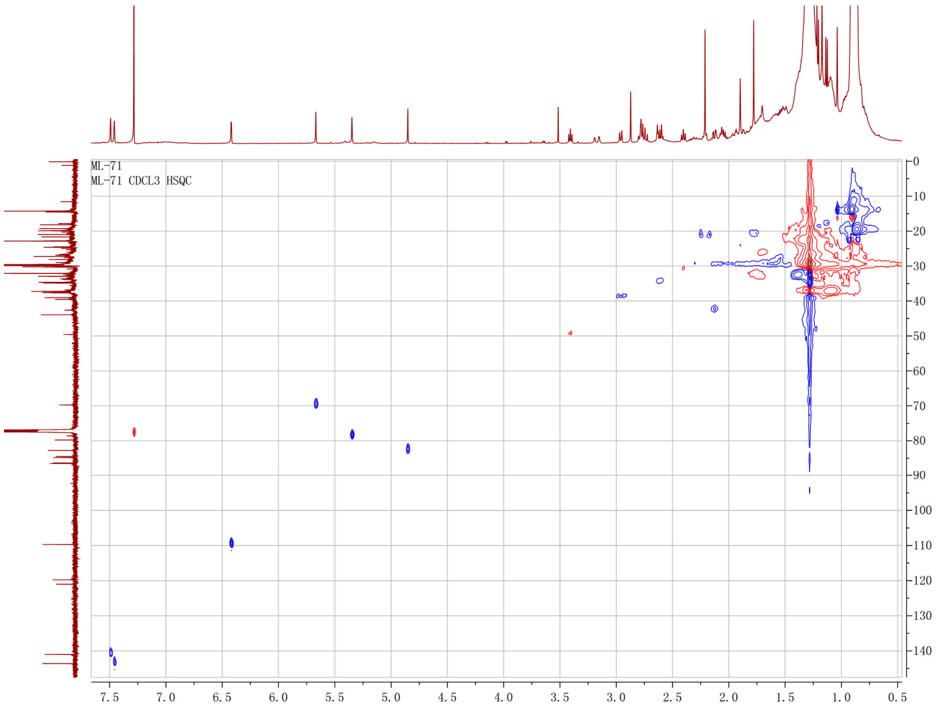

Figure S10. HSQC spectrum of the new compound **2** in $CDCl_3$.

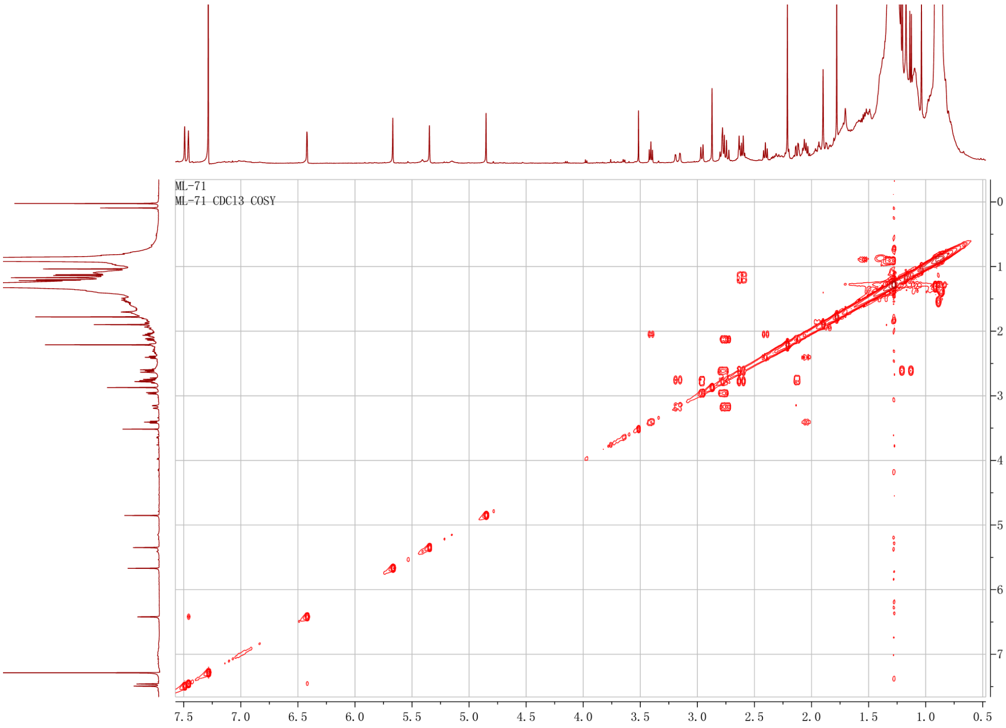

Figure S11. ^{1}H-^{1}H COSY spectrum of the new compound **2** in $CDCl_3$.

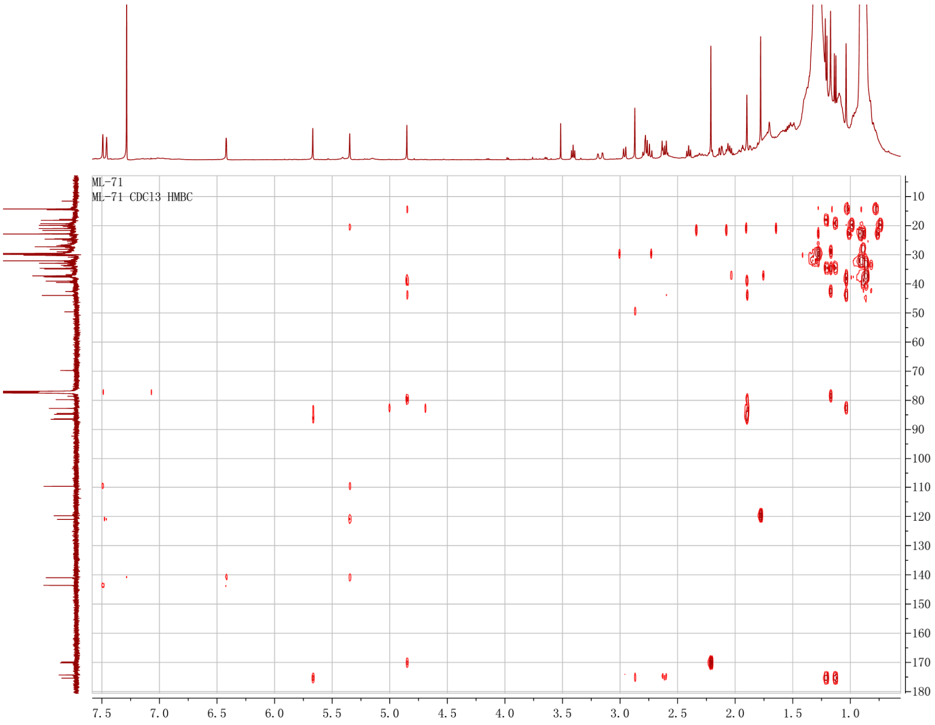

Figure S12. HMBC spectrum of the new compound **2** in $CDCl_3$.

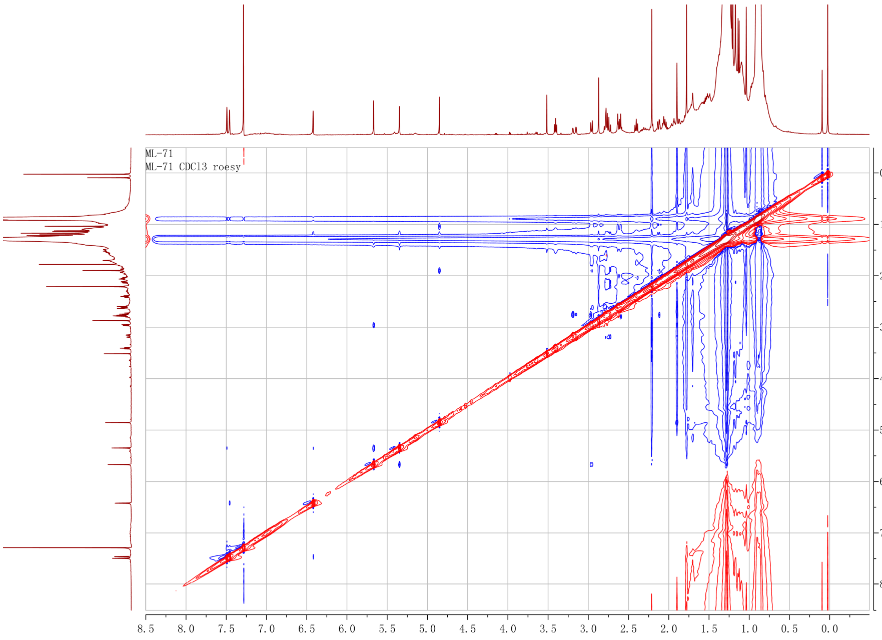

Figure S13. ROESY spectrum of the new compound **2** in $CDCl_3$.

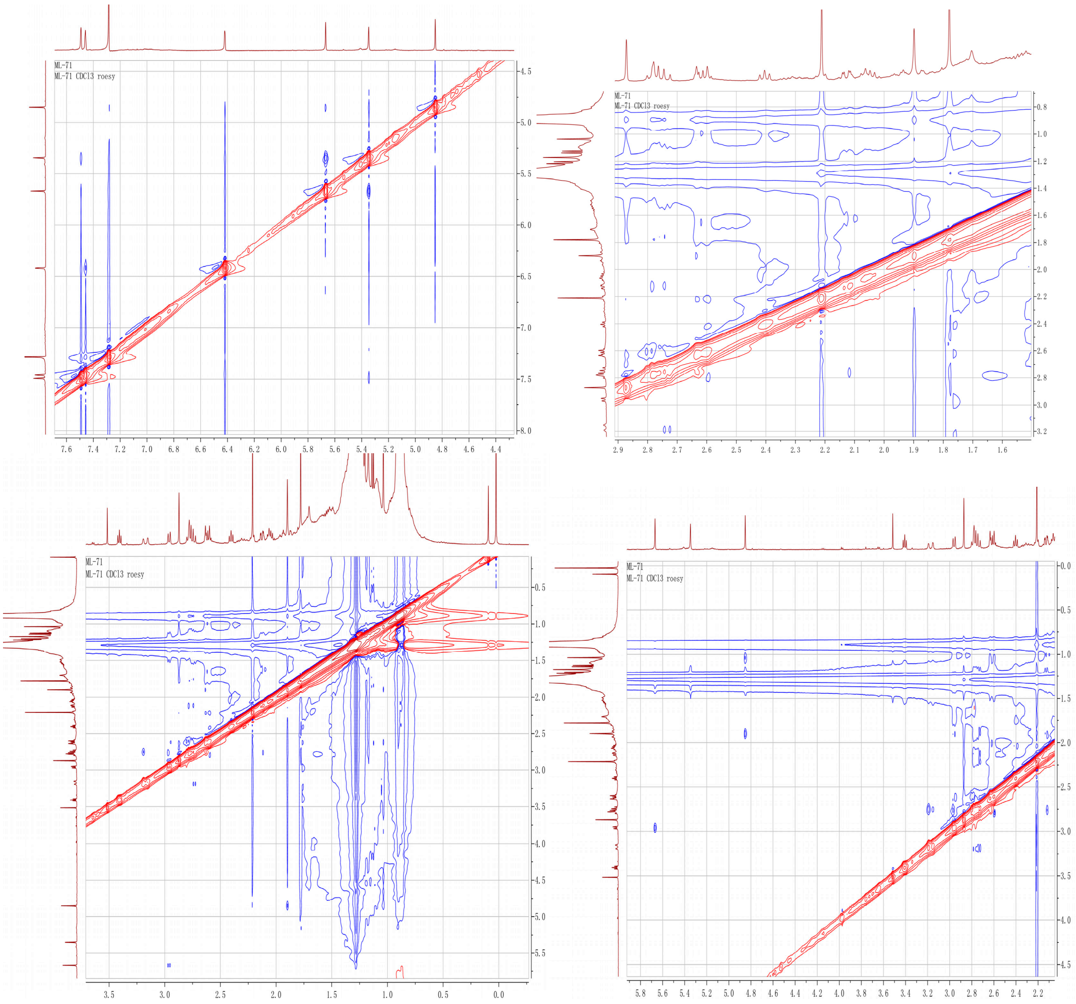

Figure S14. Partial enlarged ROESY spectra of compound **2** in $CDCl_3$.

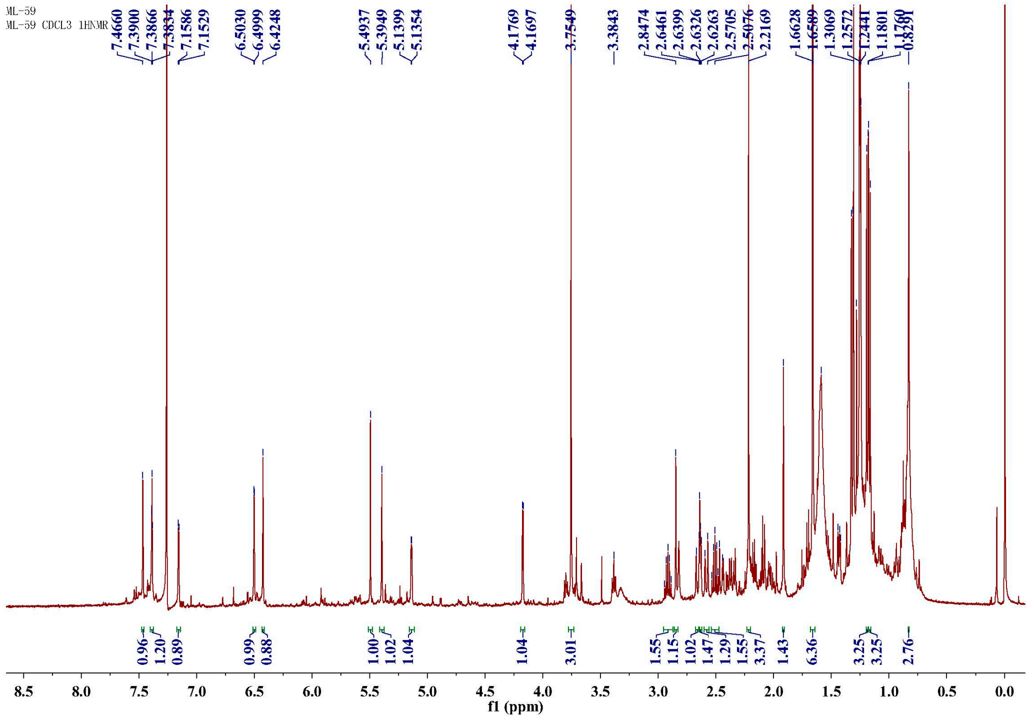

Figure S15. ^{1}H-NMR (500 MHz) spectrum of the new compound **3** in $CDCl_3$.

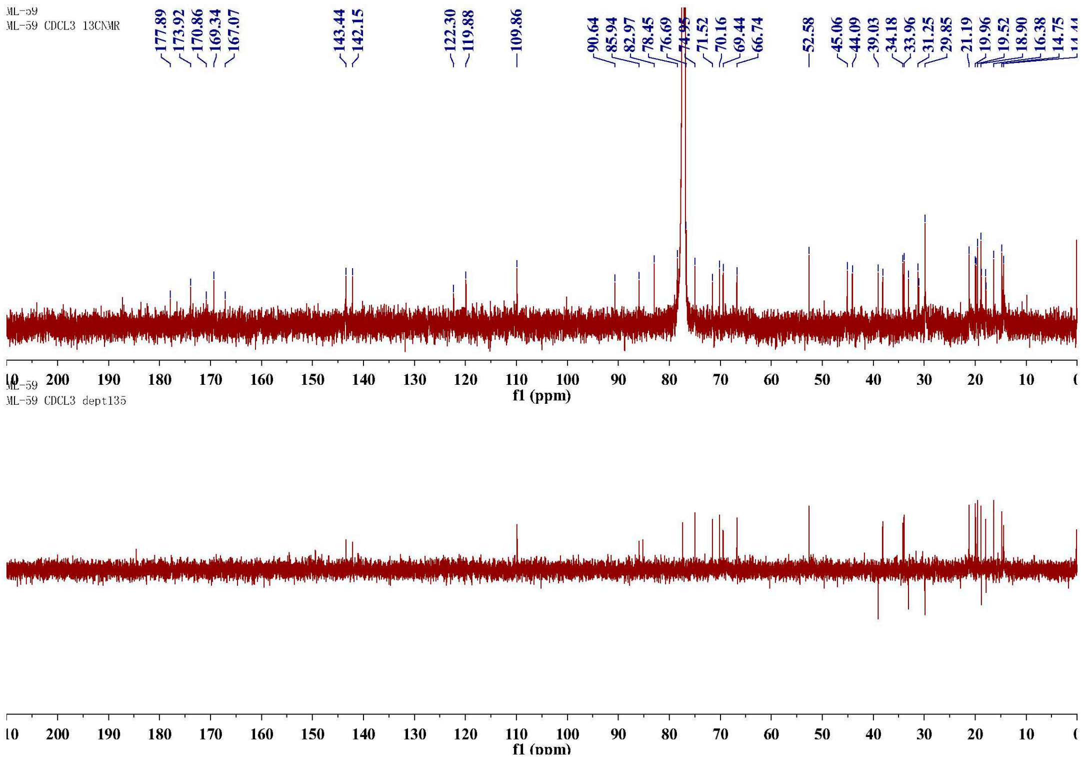

Figure S16. ^{13}C-NMR (125 MHz) and DEPT NMR spectrum of the new compound **3** in $CDCl_3$.

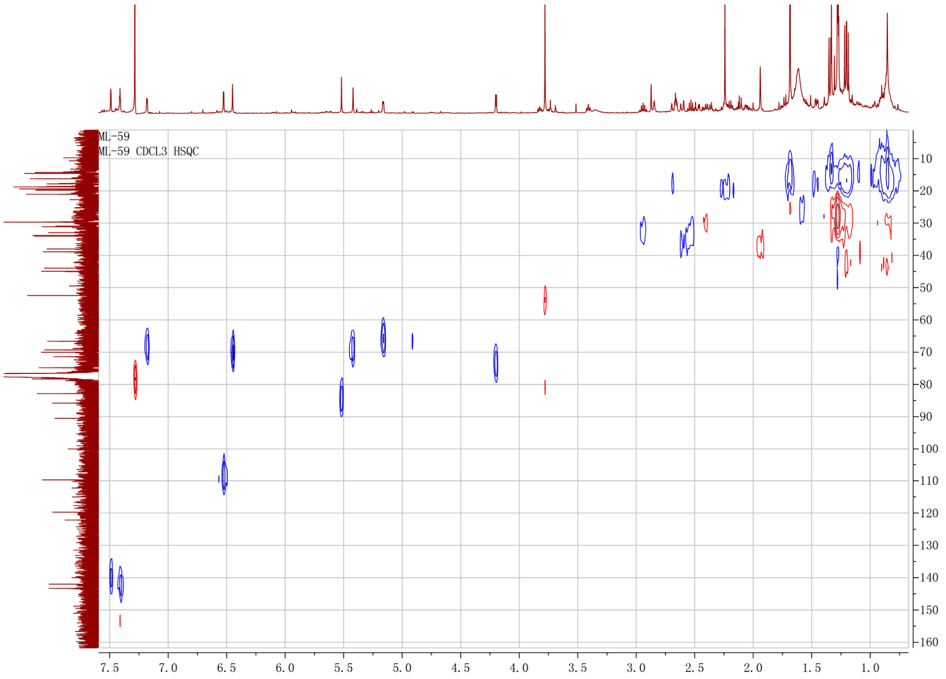

Figure S17. HSQC spectrum of the new compound **3** in $CDCl_3$.

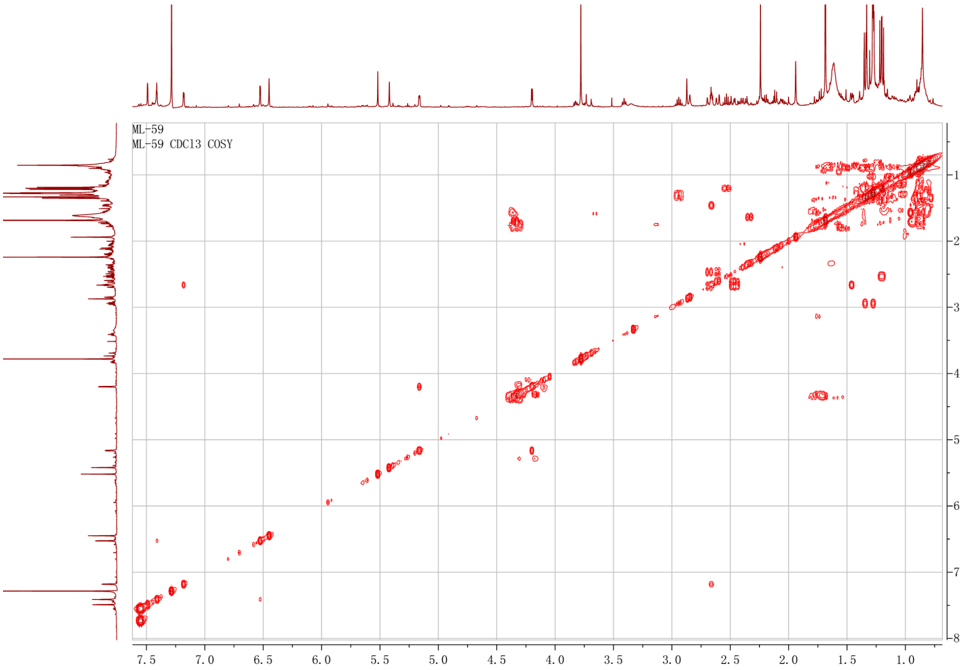

Figure S18. ^1H-^1H COSY spectrum of the new compound **3** in $CDCl_3$.

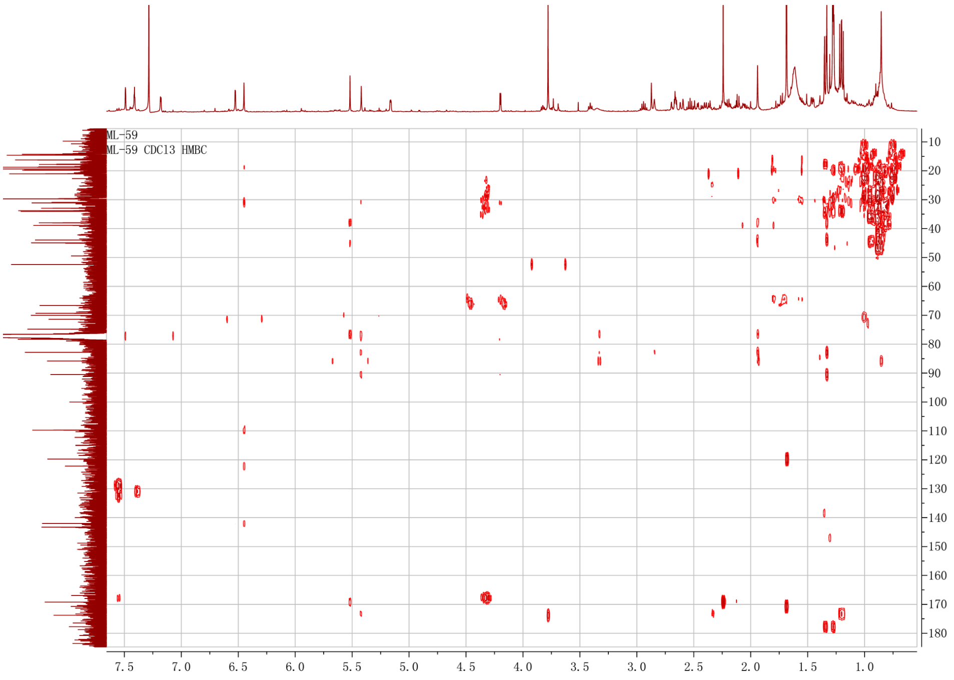

Figure S19. HMBC spectrum of the new compound **3** in $CDCl_3$.

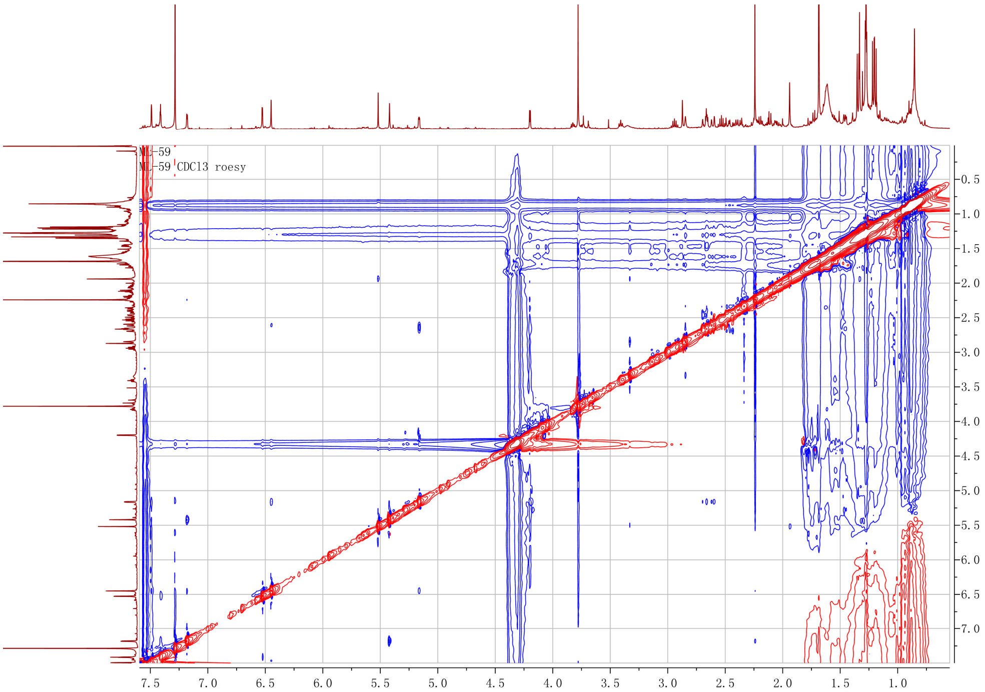

Figure S20. ROESY spectrum of the new compound **3** in $CDCl_3$.

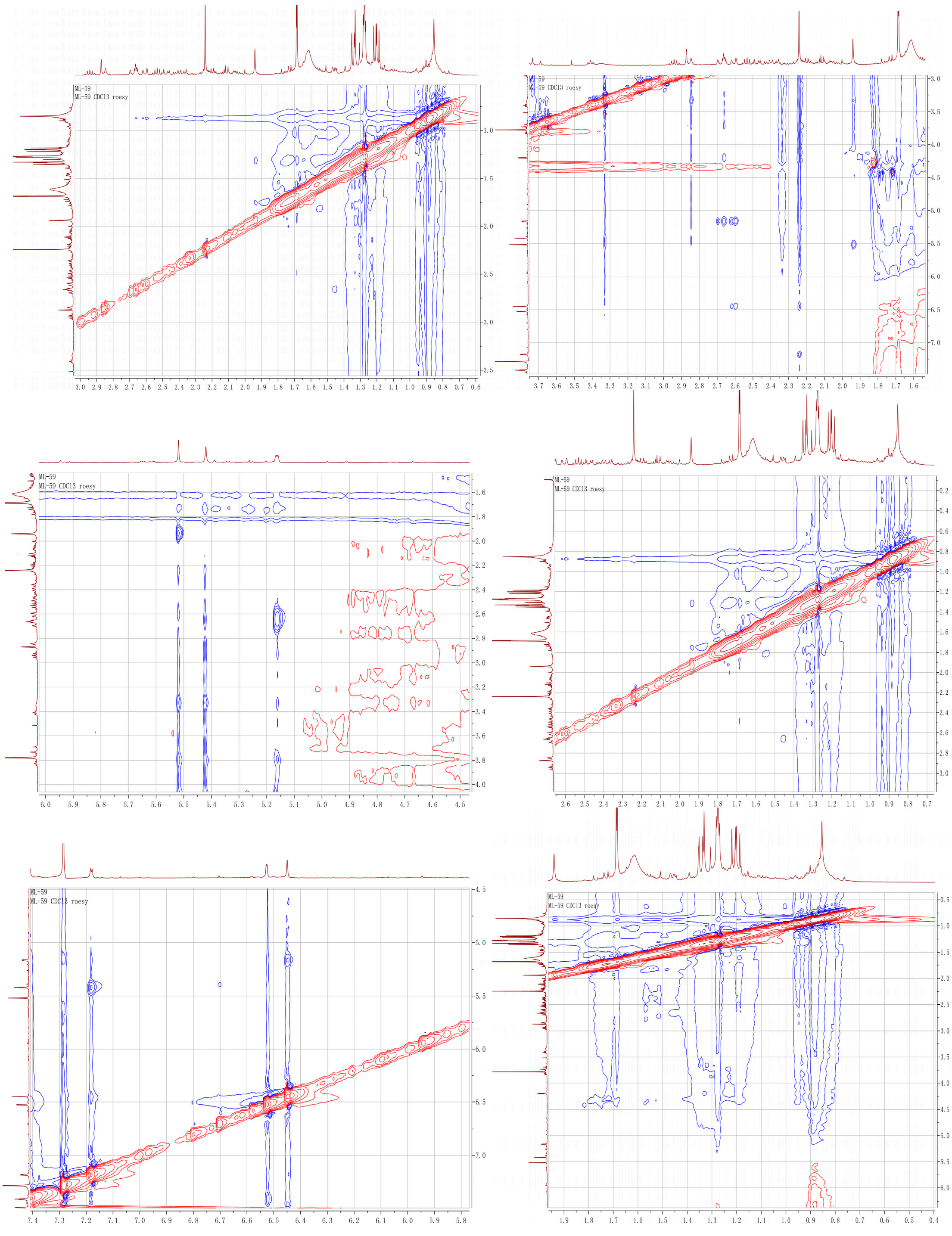

Figure S21. Partial enlarged ROESY spectra of compound **3** in $CDCl_3$.

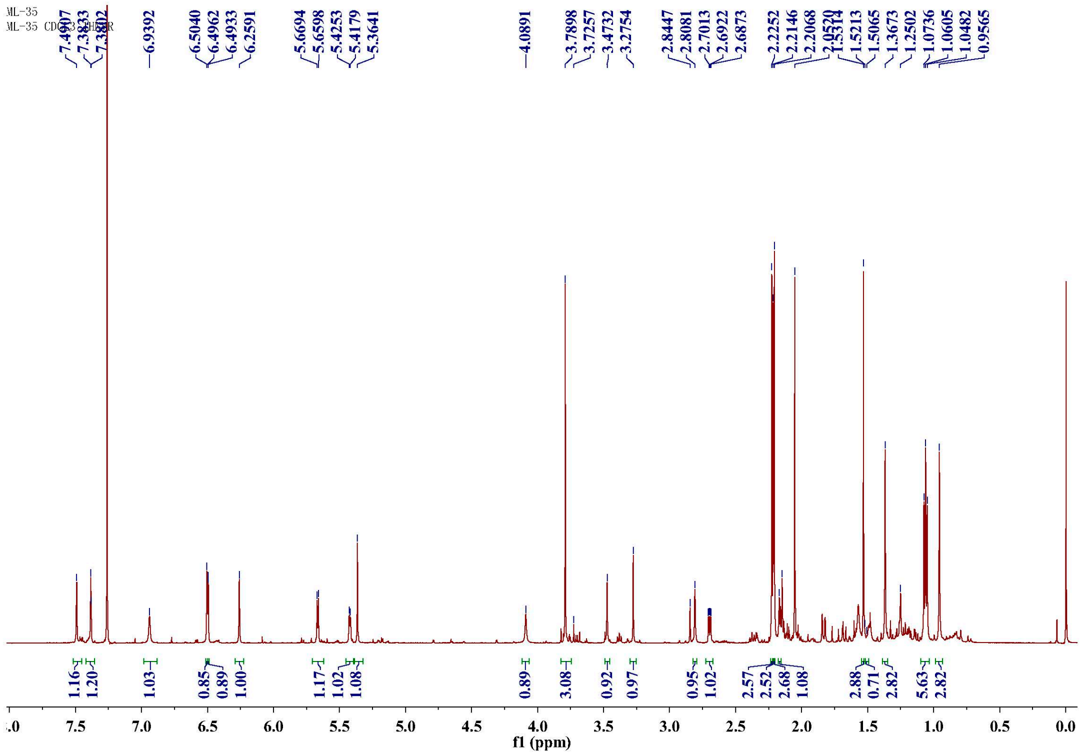

Figure S22. ^{1}H-NMR (500 MHz) spectrum of the new compound **4** in $CDCl_3$.

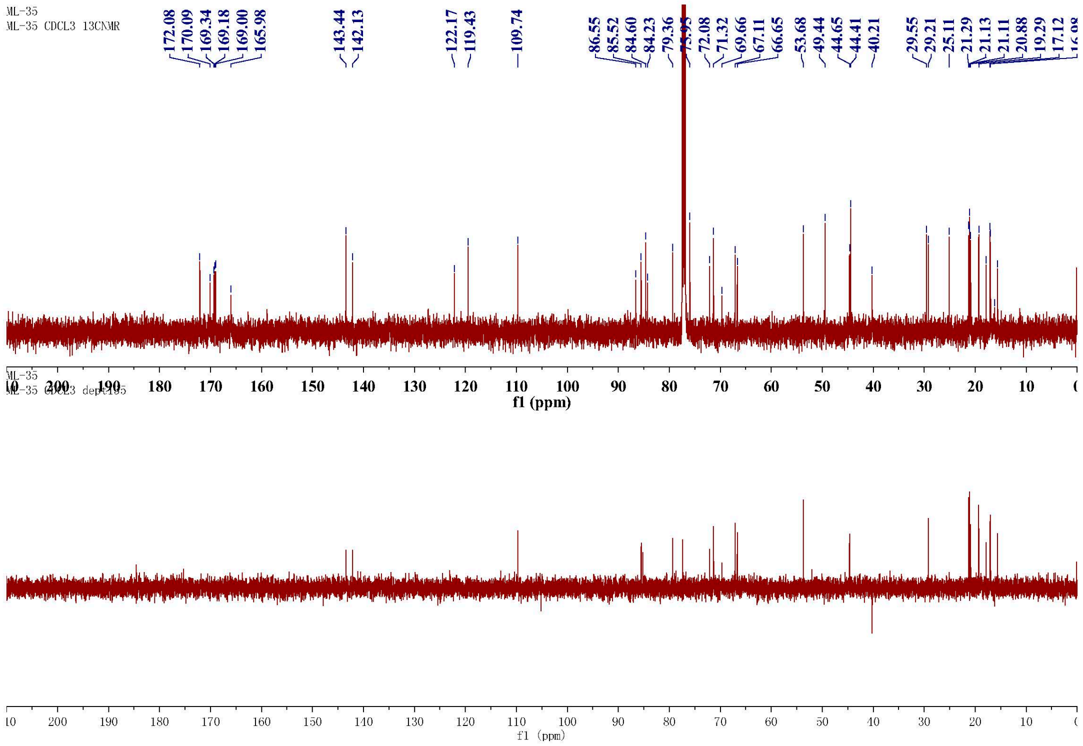

Figure S23. ^{13}C-NMR (125 MHz) and DEPT NMR spectrum of the new compound **4** in $CDCl_3$.

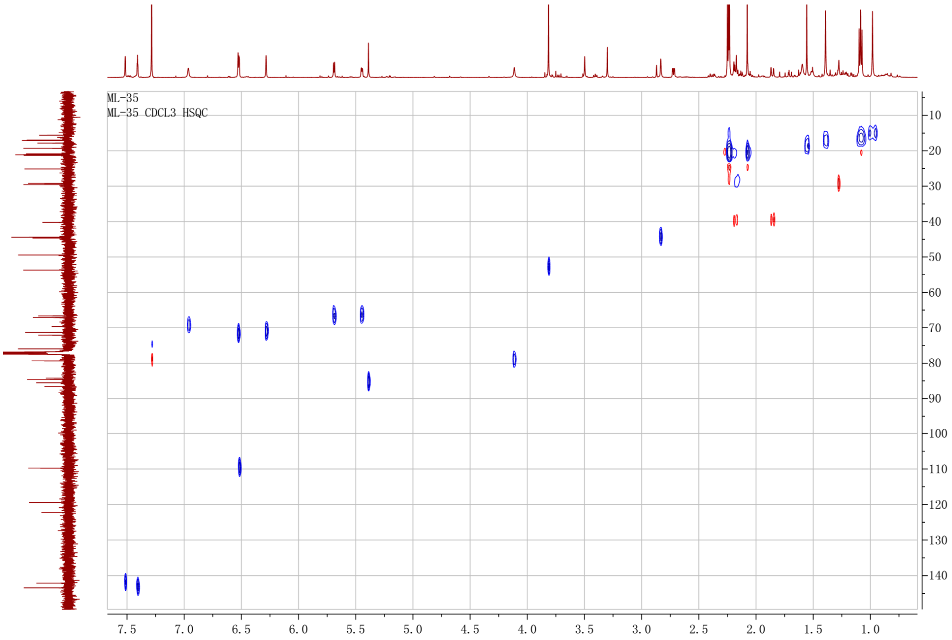

Figure S24. HSQC spectrum of the new compound **4** in $CDCl_3$.

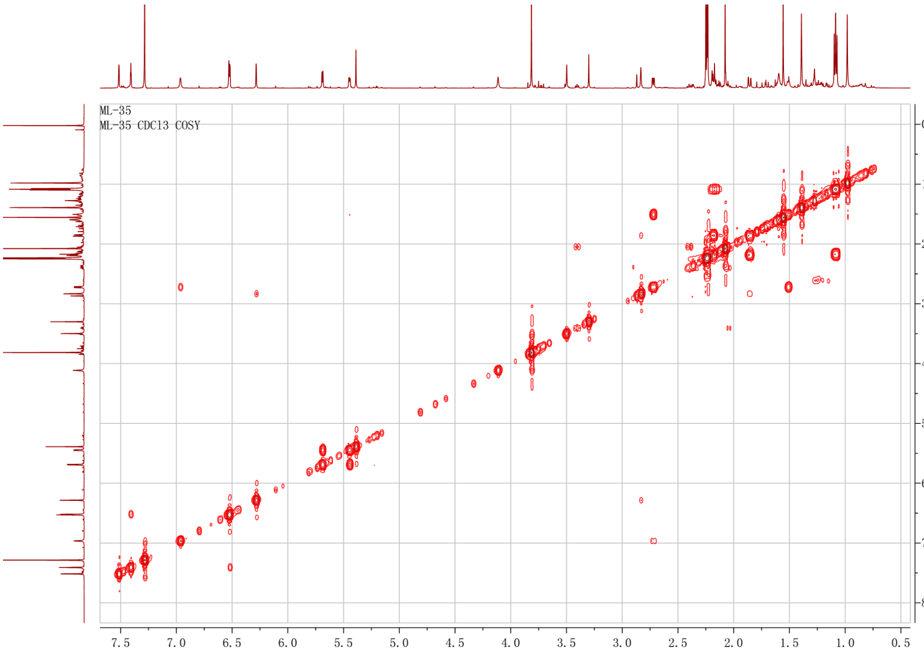

Figure S25. ^1H-^1H COSY spectrum of the new compound **4** in $CDCl_3$.

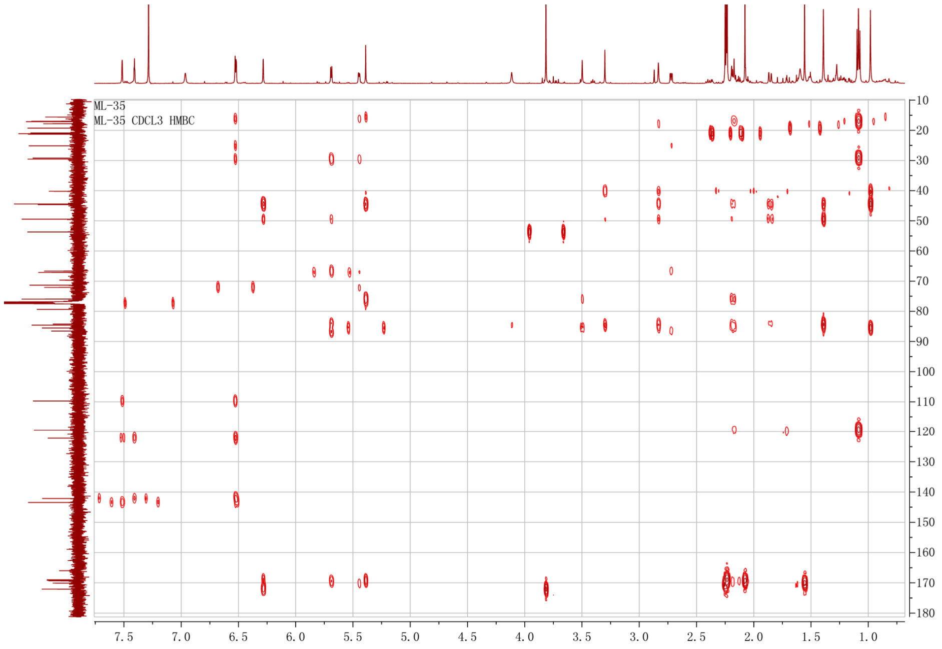

Figure S26. HMBC spectrum of the new compound **4** in $CDCl_3$.

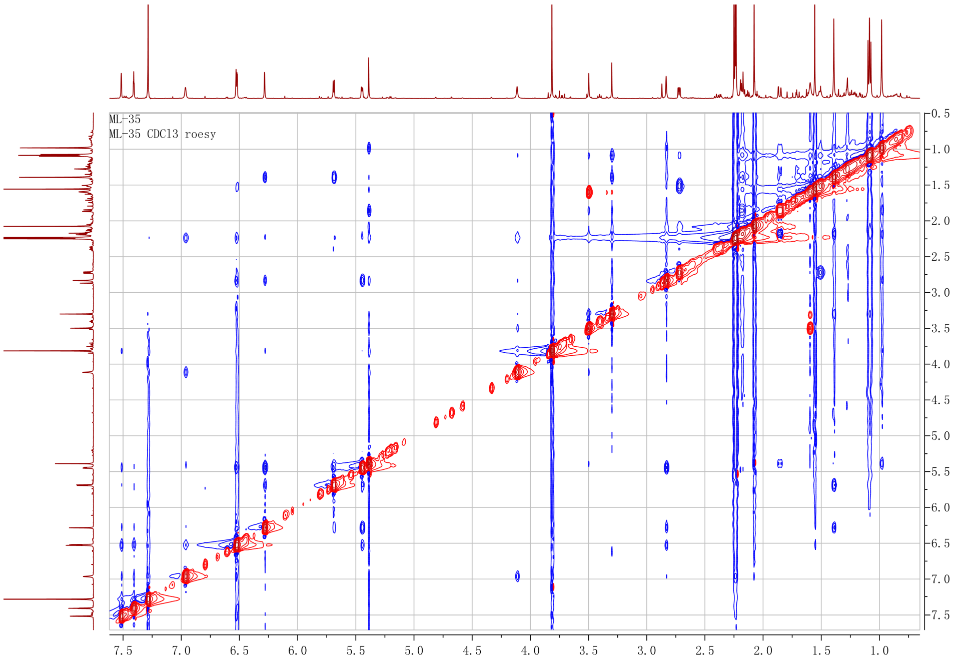

Figure S27. ROESY spectrum of the new compound **4** in $CDCl_3$.

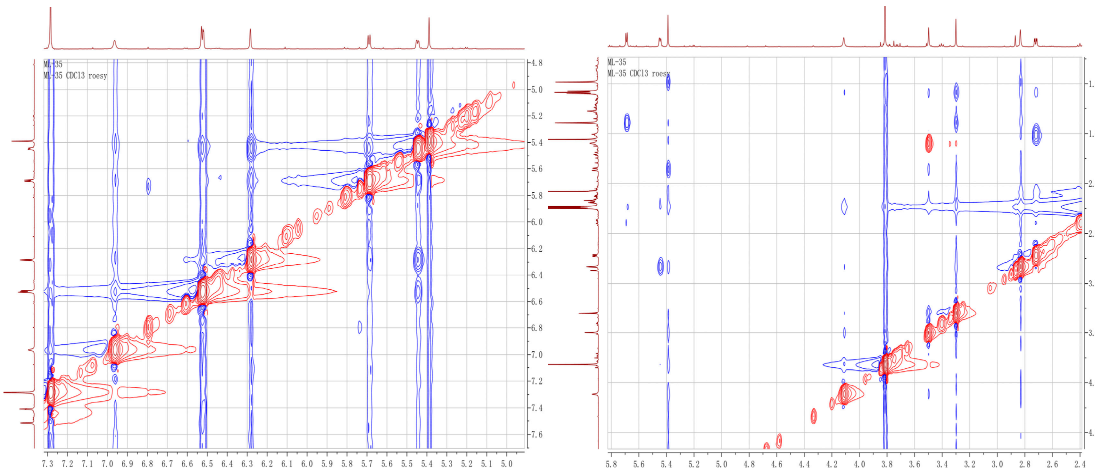

Figure S28. Partial enlarged ROESY spectra of compound **4** in CDCl3.

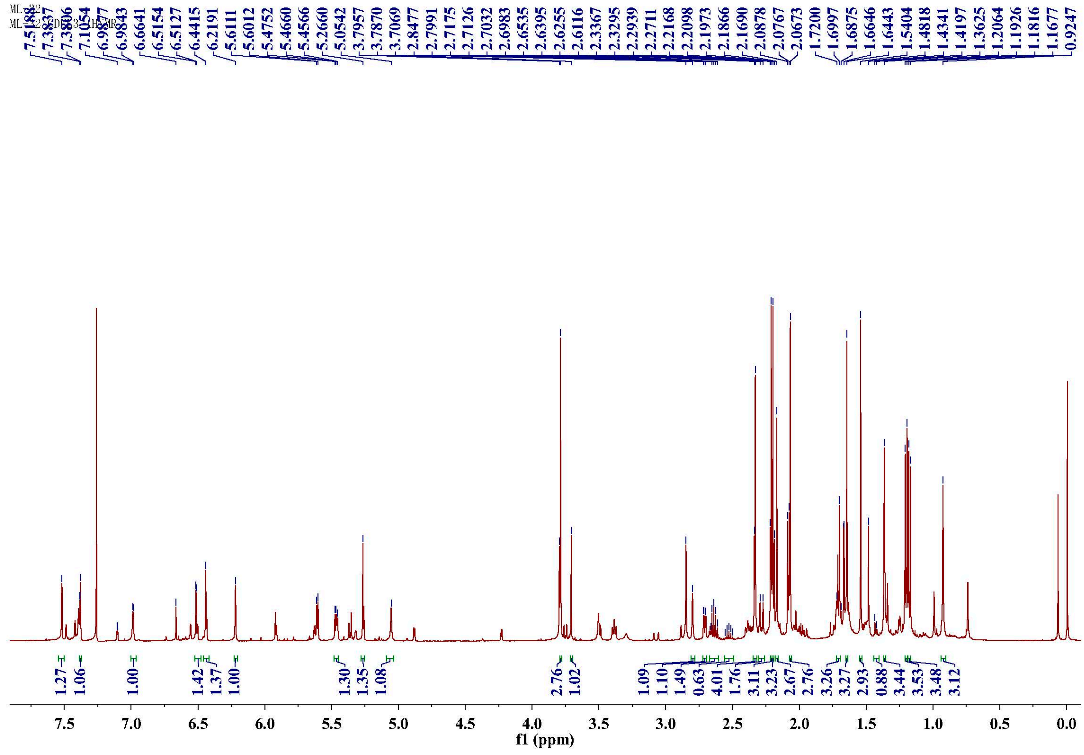

Figure S29. ^{1}H-NMR (500 MHz) spectrum of the new compound **5** in CDCl3.

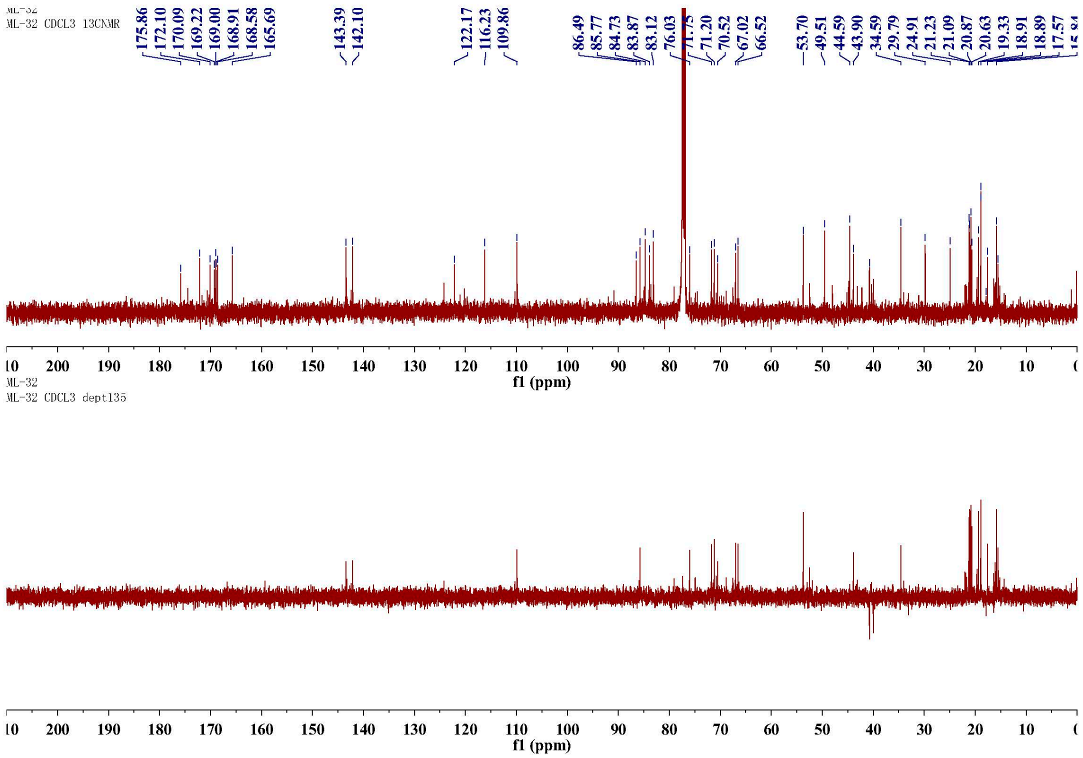

Figure S30. ^{13}C-NMR (125 MHz) and DEPT NMR spectrum of the new compound **5** in $CDCl_3$.

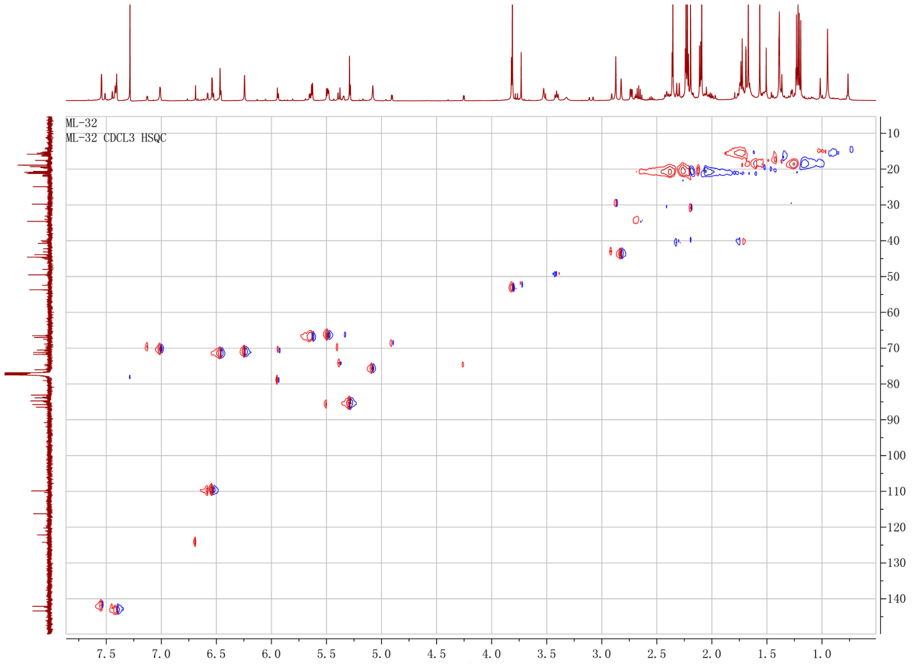

Figure S31. HSQC spectrum of the new compound **5** in $CDCl_3$.

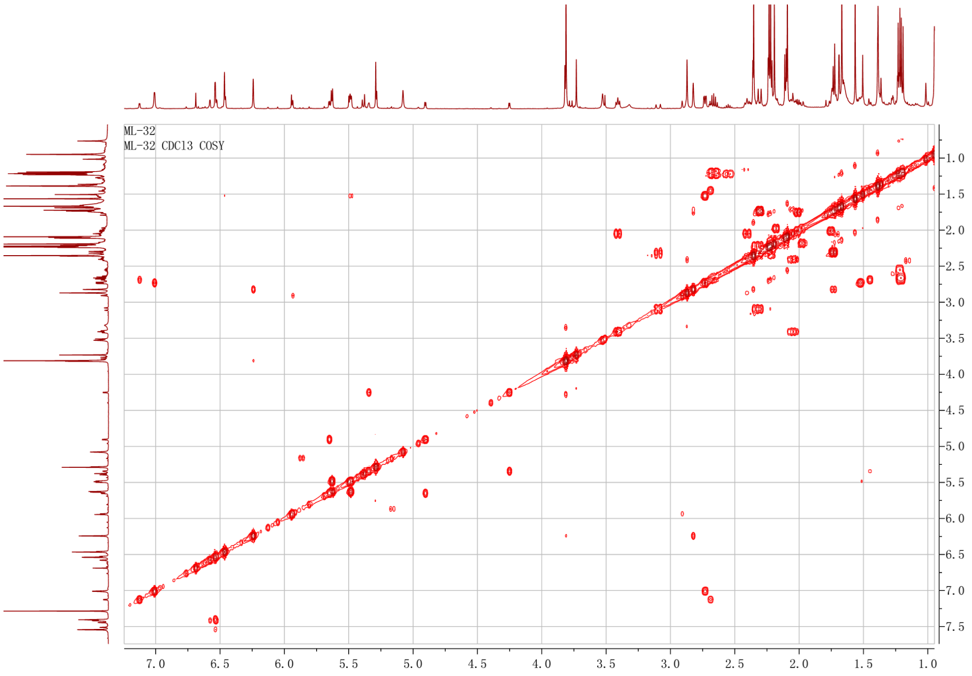

Figure S32. ^1H-^1H COSY spectrum of the new compound **5** in $CDCl_3$.

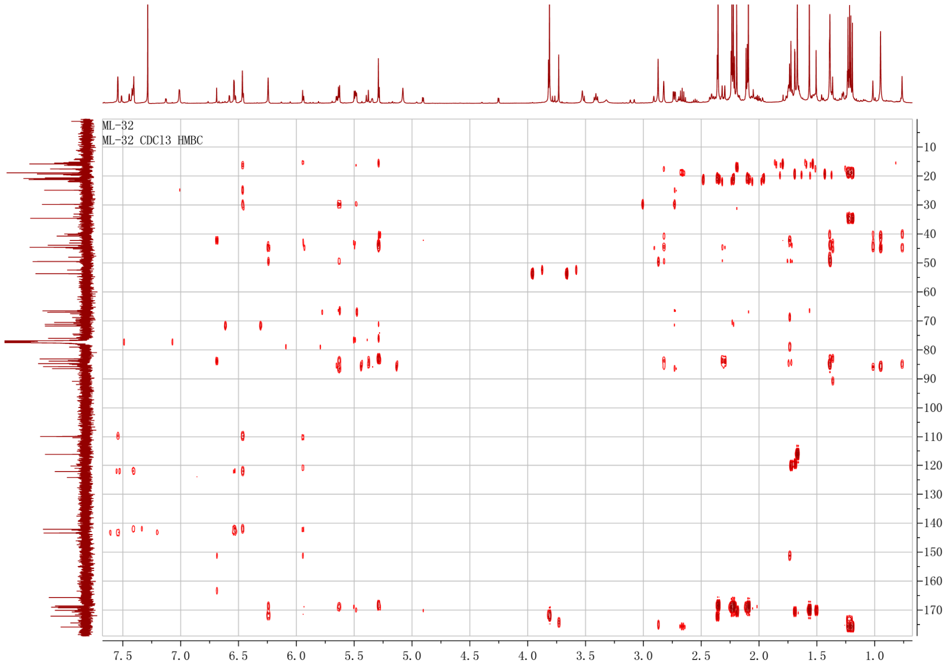

Figure S33. HMBC spectrum of the new compound **5** in $CDCl_3$.

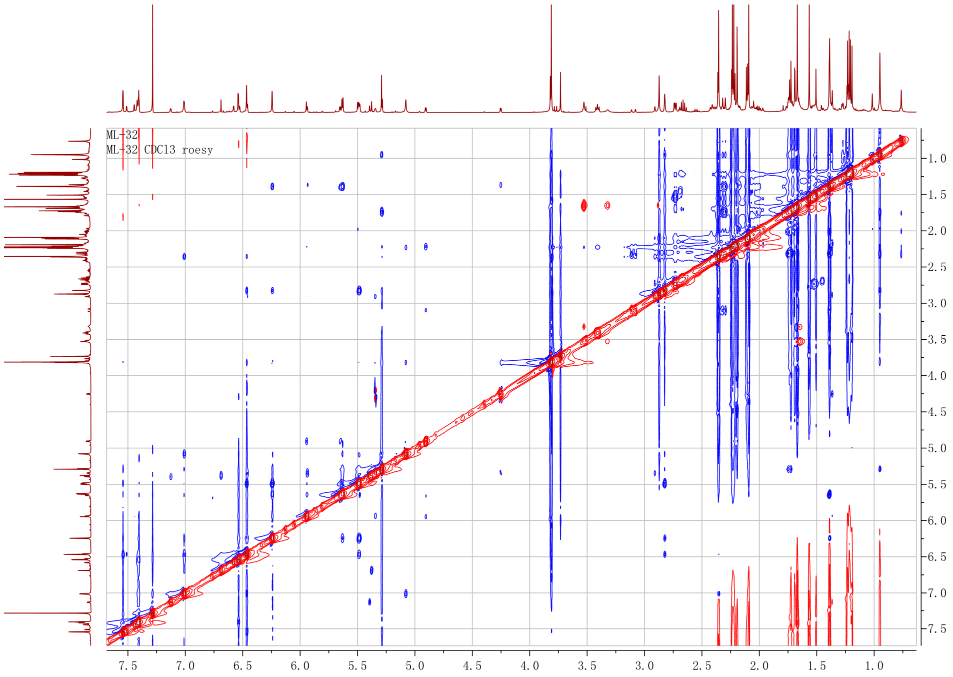

Figure S34. ROESY spectrum of the new compound **5** in $CDCl_3$.

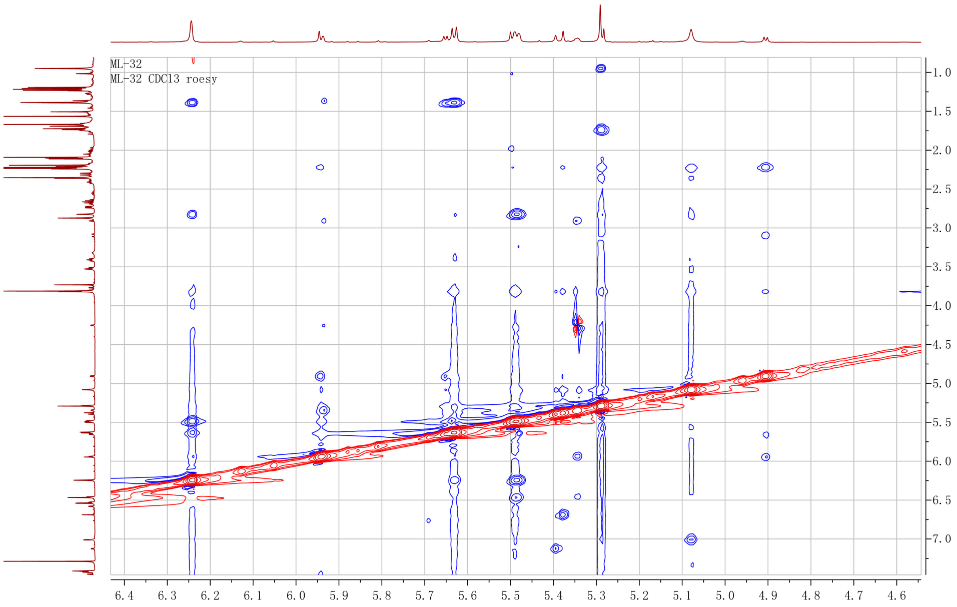

Figure S35. *Cont.*

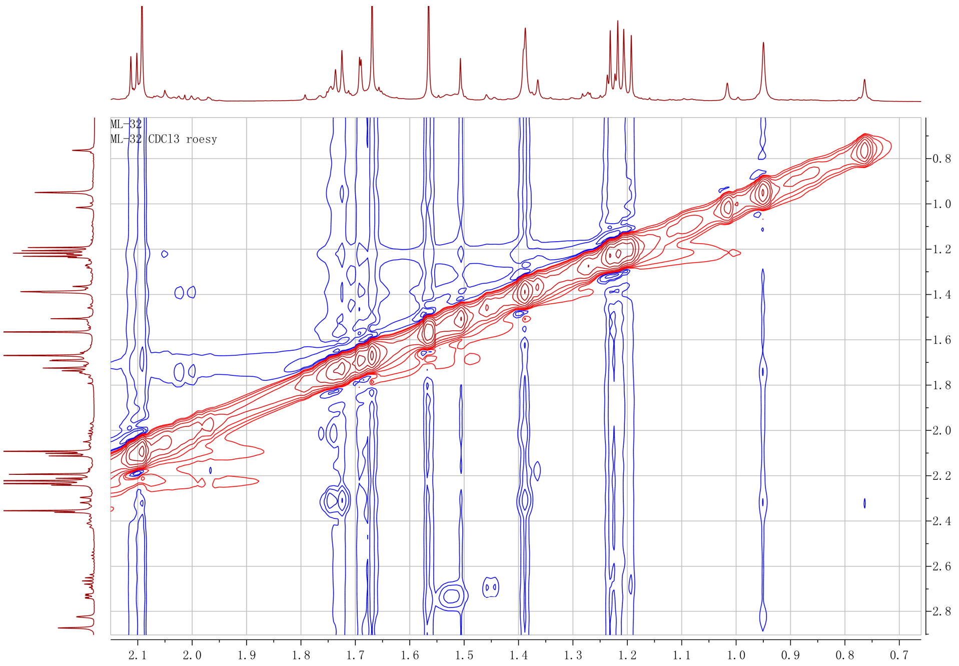

Figure S35. Partial enlarged ROESY spectra of compound **5** in $CDCl_3$.

Qualitative Analysis Report

**Data Filename**	ML-13.d	**Sample Name**	ML-13
**Sample Type**	Sample	**Position**	P1-F7
**Instrument Name**	Instrument 1	**User Name**	
**Acq Method**	SIBU.m	**Acquired Time**	7/29/2015 5:59:38 PM
**IRM Calibration Status**	Success	**DA Method**	Default.m
**Comment**			

Sample Group **Info.**
Acquisition SW 6200 series TOF/6500 series
Version Q-TOF B.05.01 (B5125.2)

User Spectra

Fragmentor Voltage	Collision Energy	Ionization Mode
135	0	ESI

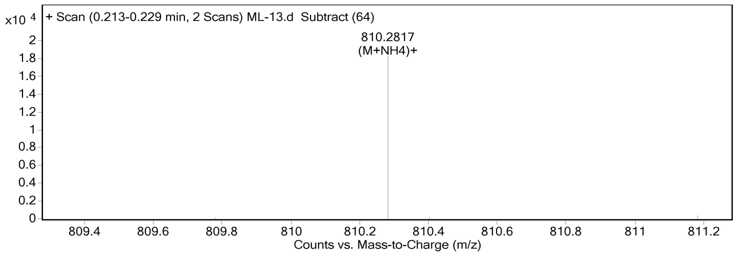

Peak List

m/z	z	Abund	Formula	Ion
318.3011		16140.07		
453.1673	1	19408.77		
460.2691	1	21296.88		
810.2817	1	18257.66	C37 H44 O19	(M+NH4)+
815.2368	1	38883.53		
816.2404	1	16098.89		
831.2107	1	15305.69		
861.3651	1	22889.48		

Formula Calculator Element Limits

Element	Min	Max
C	3	60
H	0	120
O	0	30

Formula Calculator Results

Formula	CalculatedMass	CalculatedMz	Mz	Diff. (mDa)	Diff. (ppm)	DBE
C37 H44 O19	792.2477	810.2815	810.2817	-0.1	-0.1	16.0000

--- End Of Report ---

Figure S36. HR-ESI-MS spectrum of the new compound **1**.

Qualitative Analysis Report

**Data Filename**	ML-71.d	**Sample Name**	ML-71
**Sample Type**	Sample	**Position**	P1-B9
**Instrument Name**	Instrument 1	**User Name**	
**Acq Method**	SIBU.m	**Acquired Time**	8/27/2015 3:25:11 PM
**IRM Calibration Status**	Success	**DA Method**	Default.m
**Comment**			

Sample Group **Info.**
Acquisition SW Version 6200 series TOF/6500 series Q-TOF B.05.01 (B5125.2)

User Spectra

Fragmentor Voltage	Collision Energy	Ionization Mode
135	0	ESI

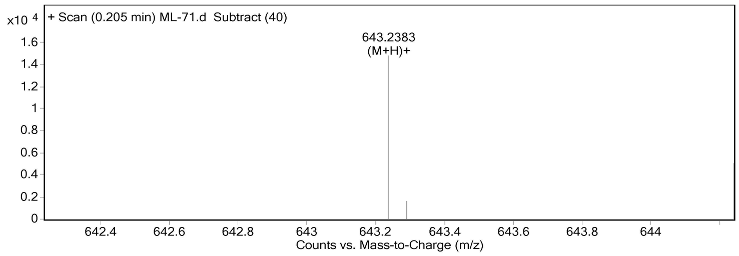

Peak List

m/z	z	Abund	Formula	Ion
318.3001		27773.34		
415.2115		16415.18		
432.238		15571.8		
437.1935	1	20423.27		
453.1673	1	54216.02		
454.1704	1	13927.89		
460.2691	1	28567.01		
643.2383	1	14764.36	C33 H38 O13	(M+H)+

Formula Calculator Element Limits

Element	Min	Max
C	3	60
H	0	120
O	0	30

Formula Calculator Results

Formula	CalculatedMass	CalculatedMz	Mz	Diff. (mDa)	Diff. (ppm)	DBE
C33 H38 O13	642.2312	643.2385	643.2383	0.1	0.2	15.0000

--- End Of Report ---

Figure S37. HR-ESI-MS spectrum of the new compound **2**.

Qualitative Analysis Report

**Data Filename**	ML-59.d	**Sample Name**	ML-59
**Sample Type**	Sample	**Position**	P1-B7
**Instrument Name**	Instrument 1	**User Name**	
**Acq Method**	SIBU.m	**Acquired Time**	8/27/2015 3:22:12 PM
**IRM Calibration Status**	Success	**DA Method**	Default.m
**Comment**			

Sample Group **Info.**
Acquisition SW Version 6200 series TOF/6500 series Q-TOF B.05.01 (B5125.2)

User Spectra

Fragmentor Voltage	Collision Energy	Ionization Mode
135	0	ESI

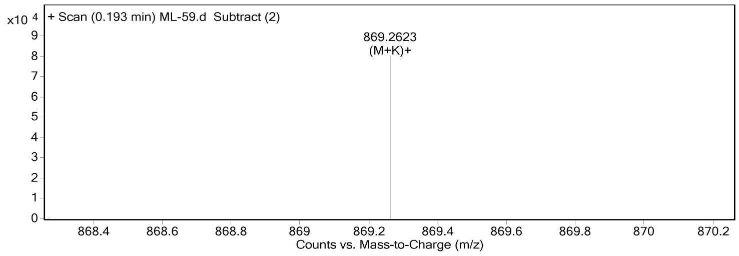

Peak List

m/z	z	Abund	Formula	Ion
274.2734	1	24594.75		
318.2999	1	25627.38		
453.167	1	20425.79		
848.3333	1	36407.78		
849.3373	1	17635.67		
853.2882	1	23147.75		
869.2623	1	80176.62	C41 H50 O18	(M+K)+
870.2657	1	37090.45	C41 H50 O18	(M+K)+

Formula Calculator Element Limits

Element	Min	Max
C	3	60
H	0	120
O	0	30

Formula Calculator Results

Formula	CalculatedMass	CalculatedMz	Mz	Diff. (mDa)	Diff. (ppm)	DBE
C41 H50 O18	830.2997	869.2629	869.2623	0.5	0.6	17.0000

--- End Of Report ---

Agilent Technologies Page 1 of 1 Printed at: 8:55 AM on: 8/28/2015

Figure S38. HR-ESI-MS spectrum of the new compound **3**.

Qualitative Analysis Report

**Data Filename**	ML-35.d	**Sample Name**	ML-35
**Sample Type**	Sample	**Position**	P1-F9
**Instrument Name**	Instrument 1	**User Name**	
**Acq Method**	SIBU.m	**Acquired Time**	7/29/2015 6:02:35 PM
**IRM Calibration Status**	Success	**DA Method**	Default.m
**Comment**			

Sample Group **Info.**
Acquisition SW Version 6200 series TOF/6500 series Q-TOF B.05.01 (B5125.2)

User Spectra

Fragmentor Voltage	Collision Energy	Ionization Mode
135	0	ESI

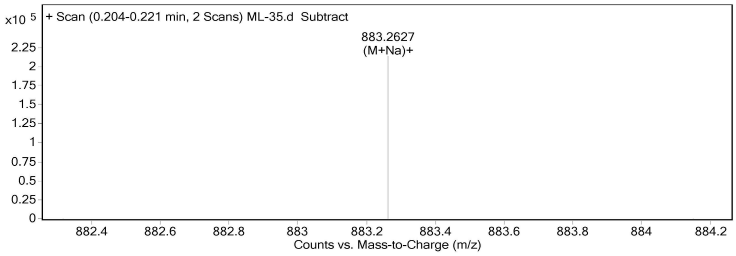

Peak List

m/z	z	Abund	Formula	Ion
878.3071	1	51829.38		
883.2627	1	214023.44	C41 H48 O20	(M+Na)+
884.2661	1	93895.03	C41 H48 O20	(M+Na)+
885.2679	1	28165.18	C41 H48 O20	(M+Na)+
899.2366	1	148595.5		
900.2401	1	65833.95		
901.2405	1	29051.7		
906.3387	1	22616.06		

Formula Calculator Element Limits

Element	Min	Max
C	3	60
H	0	120
O	0	30

Formula Calculator Results

Formula	CalculatedMass	CalculatedMz	Mz	Diff. (mDa)	Diff. (ppm)	DBE
C41 H48 O20	860.2739	883.2631	883.2627	0.5	0.6	18.0000

--- End Of Report ---

Figure S39. HR-ESI-MS spectrum of the new compound **4**.

Qualitative Analysis Report

**Data Filename**	ML-32.d	**Sample Name**	ML-32
**Sample Type**	Sample	**Position**	P1-F8
**Instrument Name**	Instrument 1	**User Name**	
**Acq Method**	SIBU.m	**Acquired Time**	7/29/2015 6:01:08 PM
**IRM Calibration Status**	Success	**DA Method**	Default.m
**Comment**			

Sample Group **Info.**
Acquisition SW Version 6200 series TOF/6500 series Q-TOF B.05.01 (B5125.2)

User Spectra

Fragmentor Voltage	Collision Energy	Ionization Mode
135	0	ESI

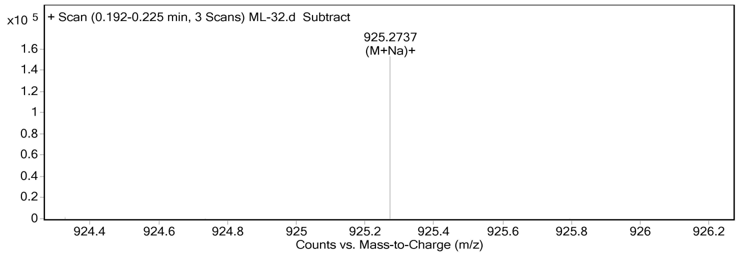

Peak List

m/z	z	Abund	Formula	Ion
899.2368	1	67953.05		
900.2403	1	31764.8		
920.3182	1	63077.2		
921.3218	1	28484.12		
925.2737	1	152962.28	C43 H50 O21	(M+Na)+
926.2772	1	67624.54	C43 H50 O21	(M+Na)+
941.2477	1	106957.15		
942.2508	1	51154.05		

Formula Calculator Element Limits

Element	Min	Max
C	3	60
H	0	120
O	0	30

Formula Calculator Results

Formula	CalculatedMass	CalculatedMz	Mz	Diff. (mDa)	Diff. (ppm)	DBE
C43 H50 O21	902.2845	925.2737	925.2737	0.0	0.0	19.0000

--- End Of Report ---

Agilent Technologies Page 1 of 1 Printed at: 3:45 PM on: 7/30/2015

Figure S40. HR-ESI-MS spectrum of the new compound **5**.
